# Plant ammonium sensitivity is associated with external pH adaptation, repertoire of nitrogen transporters, and nitrogen requirement

**DOI:** 10.1093/jxb/erae106

**Published:** 2024-03-11

**Authors:** Mikel Rivero-Marcos, Berta Lasa, Tomé Neves, Ángel M Zamarreño, José M García-Mina, Carmen García-Olaverri, Pedro M Aparicio-Tejo, Cristina Cruz, Idoia Ariz

**Affiliations:** lnstitute for Multidisciplinary Research in Applied Biology (IMAB), Sciences Department, Public University of Navarre (UPNA), Campus de Arrosadía, 31006 Pamplona, Spain; lnstitute for Multidisciplinary Research in Applied Biology (IMAB), Sciences Department, Public University of Navarre (UPNA), Campus de Arrosadía, 31006 Pamplona, Spain; CESAM—Centro de Estudos do Ambiente e do Mar, Departamento de Biologia Animal, Faculdade de Ciências, Universidade de Lisboa, 1749-016 Lisboa, Portugal; CIBIO/InBio, Centro de Investigação em Biodiversidade e Recursos Genéticos, Laboratório Associado, Instituto Superior de Agronomia, Universidade de Lisboa, Tapada da Ajuda, 1349-017 Lisbon, Portugal; Environmental Biology Department, University of Navarra, 31009 Pamplona, Spain; Environmental Biology Department, University of Navarra, 31009 Pamplona, Spain; Institute for Advanced Research in Business and Economics (INARBE), Statistics, Informatics and Mathematics Department, Public University of Navarre (UPNA), Campus de Arrosadía, 31006 Pamplona, Spain; lnstitute for Multidisciplinary Research in Applied Biology (IMAB), Sciences Department, Public University of Navarre (UPNA), Campus de Arrosadía, 31006 Pamplona, Spain; cE3c—Center for Ecology, Evolution and Environmental Changes and CHANGE, Global Change and Sustainability Institute, Faculdade de Ciencias da Universidade de Lisboa, Campo Grande Bloco C-2, 1749-016 Lisboa, Portugal; lnstitute for Multidisciplinary Research in Applied Biology (IMAB), Sciences Department, Public University of Navarre (UPNA), Campus de Arrosadía, 31006 Pamplona, Spain; Nanjing Agricultural University, China

**Keywords:** Ammonium sensitivity, ammonium transporters (AMTs), Ellenberg indicator, high-affinity nitrate transporter (NRT2), methylglyoxal, nitrogen uptake, pH adaptation, phytohormonal balance

## Abstract

Modern crops exhibit diverse sensitivities to ammonium as the primary nitrogen source, influenced by environmental factors such as external pH and nutrient availability. Despite its significance, there is currently no systematic classification of plant species based on their ammonium sensitivity. We conducted a meta-analysis of 50 plant species and present a new classification method based on the comparison of fresh biomass obtained under ammonium and nitrate nutrition. The classification uses the natural logarithm of the biomass ratio as the size effect indicator of ammonium sensitivity. This numerical parameter is associated with critical factors for nitrogen demand and form preference, such as Ellenberg indicators and the repertoire of nitrogen transporters for ammonium and nitrate uptake. Finally, a comparative analysis of the developmental and metabolic responses, including hormonal balance, is conducted in two species with divergent ammonium sensitivity values in the classification. Results indicate that nitrate has a key role in counteracting ammonium toxicity in species with a higher abundance of genes encoding NRT2-type proteins and fewer of those encoding the AMT2-type proteins. Additionally, the study demonstrates the reliability of the phytohormone balance and methylglyoxal content as indicators for anticipating ammonium toxicity.

## Teaser

This study emphasizes the importance of ecophysiological requirements and the repertoire of nitrogen transporters in understanding plant sensitivity to ammonium, and enhances our knowledge of plant nitrogen nutrition.

## Introduction

Ammonium toxicity is a universal phenomenon among organisms (e.g. for mammals, Weiner and Verlander 2011; humans, Spinelli *et al*., 2017; yeasts, Hess *et al*., 2006). In plants, the threshold for toxicity varies depending on the species, ecotype/cultivar, and environmental conditions (Britto and Kronzucker, 2002). Due to the vast number of species and ongoing crop domestication under nitrate-rich conditions, it is challenging to establish a rigid classification of ammonium-tolerant and sensitive plants. Nevertheless, general trends suggest that species thriving in acidic habitats, where ammonium is abundant, tend to be more ammonium tolerant. Conversely, plants in less acidic environments prefer nitrate and exhibit heightened sensitivity to ammonium (Britto and Kronzucker, 2002; Hawkesford *et al.*, 2012). This varying preference for ammonium or nitrate is closely linked to distinct pH tolerance. Ammonium nutrition acidifies, while nitrate nutrition alkalizes, both the external and internal plant environment. Ammonium uptake by roots results in a net efflux of protons (H^+^) to the cell to maintain membrane potential, and its assimilation into glutamine generates H^+^, thus acidifying the rhizosphere and the cellular environment, especially in the shoot tissue (Raven and Smith, 1976; Feng *et al*., 2020; Hachiya *et al*., 2021). Conversely, nitrate uptake involves co-transport with H^+^, resulting in a net influx of H^+^ into the roots. This H^+^ influx into the plant is then offset by the H^+^-consuming nitrate reduction in the shoot, and the release of organic acids such as malate, to maintain the acid–base balance and regulate the cytosolic pH in the cell. Consequently, nitrate nutrition has a net alkalizing effect on the rhizosphere and within the plant (Raven and Smith, 1976; Feng *et al*., 2020).

While existing studies predominantly explore metabolic, hormonal, or genotypic aspects of plant responses to ammonium and nitrate (e.g. Zhu *et al.*, 2006; Guo *et al.*, 2007; Patterson *et al.*, 2010; Engelsberger and Schulze, 2012; Dechorgnat *et al.*, 2019), the ecological and physiological traits facilitating nitrogen uptake and adaptation receive comparatively less attention. Given the diversity in sensitivity to ammonium among plant species, it is crucial to have a comprehensive understanding of ecophysiological features enabling nitrogen acquisition in different habitats. Ellenberg indicator values (EIVs) are a useful tool for numerically classifying the habitat niches and occurrence peaks of plant species along gradients such as light availability, temperature, continentality, soil moisture, soil pH (R), soil fertility or productivity based on nitrogen demand (N), and salinity (Ellenberg *et al.*, 1992; Schaffers and Sýkora, 2000). Correlating EIVs with relevant traits can help identify factors influencing species distributions, including soil pH, ammonium tolerance, or nitrogen use efficiency, which in turn are primarily determined by root capacity to take up nitrate and ammonium.

The root system plays a pivotal role in the initial encounter with environmental ammonium and nitrate, which necessitates a concurrent initiation of uptake, transport, and assimilation across plant parts (Masclaux-Daubresse *et al.*, 2010). In fact, different changes in the root system architecture can be triggered by environmental nitrate and ammonium. These localized responses to nitrogen in the root system depend on the coordination of internal and environmental signals, some of which are mediated by nitrogen transporters, and may result in differential acquisition of ammonium or nitrate (Giehl *et al.*, 2014).

In 2014, von Wittgenstein *et al.* conducted a phylogenetic analysis of nitrogen transport families in land plants that revealed the early divergence of high-affinity ammonium transport families (AMT1 and AMT2) before the separation of bryophytes and vascular plants. Furthermore, the high-affinity nitrate transport family (NRT2) diverged early in the evolution of vascular plants, while the low-affinity system for nitrate (NRT1/PTR) evolved later in the remaining land plants (von Wittgenstein *et al*., 2014). The evolution of vascular plants resulted in a higher concentration of oxygen in the atmosphere and altered the nitrogen cycle, promoting ammonium oxidation to nitrate (Bendall *et al.*, 2008). This provided modern crop plant ancestors with mechanisms for increased nitrate acquisition through the polyphyletic NRT1/PTR family.

Given the lack of relationship between specific soil conditions (such as pH or cation–anion availability) and plant nitrogen uptake systems, this study aims to identify determining factors that explain plant nitrogen uptake capacity and sensitivity to the two major inorganic nitrogen forms, nitrate and ammonium. In order to achieve this objective, and given that soil acidification causes higher and lower availability of ammonium and nitrate, respectively, the hypothesis to be tested is that ‘the evolutionary adaptation of plants to soil conditions may be linked to the number of each type of AMT and NRT transporter, affecting optimal nitrogen uptake’. Consequently, the distinct repertoire of nitrogen transporters in each species may indicate a ‘preference’ for the primary nitrogen form in certain soils and, in turn, the degree of sensitivity to ammonium. To test the proposed hypothesis, firstly, a comprehensive meta-analysis of 68 studies from 1967 to 2022 will explore the comparative growth of various plant species under nitrate or ammonium nutrition. Then, this biomass-based classification of plant species will be correlated with intrinsic EIVs of their habitats and *in silico* analysis of their nitrogen transport systems. Finally, the classification will be tested by a comparative analysis of developmental and metabolic responses, using hormonal balance as a metabolic indicator, in two species that are divergent from each other in the proposed classification.

For the first time, this study proposes a basis for classifying plant species based on their sensitivity to ammonium, addressing three key questions: (i) association between ecophysiological factors and nitrogen preference of plant species; (ii) difference in ammonium uptake capacity between tolerant and sensitive species; and (iii) prediction of ammonium sensitivity by hormonal and metabolic indicators.

## Materials and methods

### Meta-analysis of nitrogen nutrition experiments

#### Data search and selection criteria

Data were extracted from studies where growth and other physiological parameters of ammonium-fed plants could be compared with those of nitrate-fed plants under equivalent conditions. A survey of peer-reviewed published literature was conducted to identify articles that reported the growth of plants with ammonium or nitrate using the academic literature database ISI Web of Science, from January 1967 to March 2022.

**Fig. 1.**
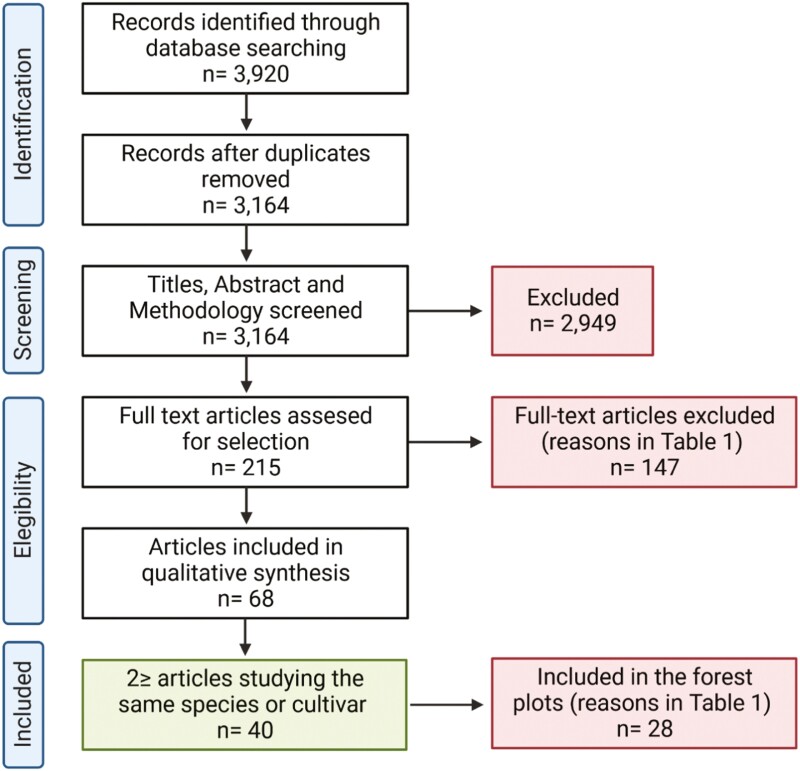
Workflow performed for the selection of the number of studies to be retained and discarded at each stage of the systematic literature review.

The following Boolean search string was used in each database: Topic=(‘Nitrogen Nutrition’) OR (‘Ammonium Tox*’) OR (‘Nitrogen Form’); refined by research areas=(‘Plant Sciences’ OR ‘Agriculture’ Or ‘Environmental Sciences Ecology’), which yielded ~3164 results after duplicates were removed. Titles, abstracts, and materials and methods were vetted by two reviewers (MR and TN), and the Kappa statistic was used to evaluate inter-reviewer agreement (Kappa=0.94: near-perfect level of agreement; Fig. 1). We retrieved and reviewed 215 full-text articles against inclusion and critical appraisal criteria (Table 1). An important part of the first inclusion criterion was the use of a hydroponic cultivation system, which ensures controlled conditions for nutrient availability and homogeneous and standardized exposure to nutrients. DataThief III was used to collect the data if they were only present in graphical format. After full-text screening, quantitative data were extracted from 68 studies and considered for being included in a meta-analysis.

**Table 1.** Criteria for study inclusion and critical appraisal during quantitative synthesis

Inclusion criteria	Critical appraisal criteria
Primary study including a quantitative comparison of root and/or shoot **biomass** of plant species grown in **hydroponics** with **nitrate versus ammonium** as the sole source of nitrogen.	Non-hydroponic cultivation or insufficient information on the nutrient solution (e.g. pH value).
The study provides an exact *P*-value or a statistical result (*Z*, *F*, *t*, *r*, *r*^2^, or χ^2^) accompanied by the sample size (minimum of three biological replicates) and the SD or SE. Alternatively, the study can provide raw data on biomass under ammonium or nitrate nutrition.	No nitrate or ammonium control treatment. Study does not report error values or sample sizes.
The meta-analysis comprised at least two studies discussing the same species or cultivar to strengthen sample size and statistical reliability.	Parameters are manually included in forest plot graphics for species and cultivars with a single study, as long as they meet the above requirements, aiming to obtain a rough understanding of the ammonium response between specific cultivars.

#### Data processing and total biomass calculation

(i) In studies where only shoot and root biomass were shown, total biomass from shoot biomass and root biomass was calculated with the following equations and assumptions:


$$\begin{array}{l} {\bar{X}}_{Plant\;Biomass}={\bar{X}}_{Shoot\;Biomass}+{\bar{X}}_{Root\;Biomass} \end{array}$$

$$\begin{array}{l} {\mathrm{var}}_{Plant\;Biomass}={\mathrm{var}}_{Shoot\;Biomass}+{\mathrm{var}}_{Root\;Biomass}+2{\mathrm{cov}}_{Root-shoot\;Biomass} \end{array}$$

For this last calculation and based on the data gathered from the studies, the following assumption was taken: ‘biomass of root and shoot are independent variables’, thus 2cov_Root–Shoot Biomass_ could be considered minimal or even 0. Thus,


$$\begin{array}{l} 2{\mathrm{cov}}_{Root-shoot\;Biomass}∼0 \end{array}$$

(ii) In articles studying vegetative and first phenological states of plant species with shoots in rosettes, leaf biomass has been considered as shoot biomass.

(iii) In studies where leaf biomass and stem biomass were considered separately, a similar procedure to that shown in point (i) for biomass and variance calculations and assumptions was taken on:


$$\begin{array}{l} {\bar{X}}_{Shoot\;Biomass}={\bar{X}}_{Stem\;Biomass}+{\bar{X}}_{Leaf\;Biomass} \end{array}$$

$$\begin{array}{l} {\mathrm{var}}_{Plant\;Biomass}={\mathrm{var}}_{Shoot\;Biomass}+{\mathrm{var}}_{Root\;Biomass}+2{\mathrm{cov}}_{Root-shoot\;Biomass} \end{array}$$

$$\begin{array}{l} 2{\mathrm{cov}}_{Root-shoot\;Biomass}∼0 \end{array}$$

#### Size effect calculation

For each study, the natural log-transformed response to the nitrogen source ratio (lnBR) was used as a measure of effect size (Hedges *et al.*, 1999):


$$\begin{array}{l} lnBR=ln\left(\;\;\;\frac{{\bar{x}}_{NH4+}}{{\bar{x}}_{NO3}\text{-}}\right) \end{array}$$

where ${\bar{x}}_{NH4+}$ is the mean of the plants’ organ biomass (i.e. root or shoot) grown in ammonium, and ${\bar{x}}_{NO3\text{\textasciitilde{}}\text{-}}$ is the mean of those grown in nitrate. To calculate the variance, the following approximation was used (Hedges *et al.*, 1999):


$$\begin{array}{l} var_{lnBR\;\;\;}=\frac{{(SD_{NH4})}^{2}}{N_{NH4}{({\bar{x}}_{NH4})}^{2}}+\frac{{(SD_{NO3})}^{2}}{N_{NO3}{({\bar{x}}_{NO3})}^{2}} \end{array}$$

#### Meta-analysis

All analyses were performed using Metawin 2.1 (https://www.metawinsoft.com).

Depending on the analysis, both categorical and continuous models were used; however, as a variety of species with different growth conditions were analyzed, a random effects model was always chosen, with the study as the random effect.

In order to perform the meta-analysis, at least two studies meeting all data selection criteria for each group were required. This cut-off point resulted in 40 studies being included in the meta-analysis (considering 21 plant species), and cases where only one study was available being excluded from the meta-analysis (i.e. 28 studies and consequently 29 plant species; Fig. 1; Table 1). Nevertheless, these 28 single studies were still included in the figures, which are duly marked in the figures, in order to obtain a rough understanding of the ammonium response between plant species and thus enriching this first proposal for plant species classification.

Effect sizes were considered significantly different from 0 if their 95% confidence intervals did not overlap, and differences between groups were considered significant if their 95% confidence intervals did not overlap. Confidence intervals based on variance were considered (SD×1.96).

The Rosenthal method (Rosenthal, 1979) was used to account for the possibility of publication bias. Depending on the analysis, values between 350 and 2000 were obtained. As these values were always at least more than twice the number of studies used, it is unlikely that the results were affected by publication bias.

The cases included in the meta-analyses were weighted according to their relevance and size, namely the number of biological replicates (number of each species) and number of studies.

#### Global indicators of the significance of meta-analyses, and cross-validation analysis

Means of pooled effect sizes (i.e. LnBR values) for each condition (organ and pH) were calculated as global indicators of significance for each meta-analysis performed.

As a cross-validation analysis, bootstrap analyses were performed with 1000 iterations each and confidence intervals were recalculated as a result. As expected, the bootstrap confidence intervals were consistently smaller than those calculated from variance (Supplementary Dataset S1). However, those obtained from the variance (SD×1.96) are shown in the figures because starting with the ‘worst case’ and showing the largest gives more robustness to the results. Showing the variance confidence intervals also helps to maintain consistency with the other 29 plant species (individual studies) that were manually added to the figures.

### Ellenberg indicator values for nitrogen and soil pH

Using the databases ‘Global Biodiversity Information Facility’ (GBIF; https://www.gbif.org/) and ‘Plant Database Search’ (PFAF; https://pfaf.org/user/Default.aspx), together with the EIVs (Ellenberg *et al.*, 1992), we analyze the environmental factors and ecological requirements of the plant species from the meta-analysis in terms of soil pH (R), nitrogen requirement (N), and the number of countries in which a certain species can be invasive (spread capacity). It is important to point out that the EIVs describe N indistinctly as nitrogen requirement, soil fertility, nitrophily, or even productivity (Schaffers and Sýkora, 2000), something that in most soils is conferred by and related to nitrate soil content, as the main source of nitrogen.

### Genes encoding nitrogen transporters in different plant species

The ‘Arabidopsis Information Resource’ (TAIR) database (http://www.arabidopsis.org/, accessed 2 February 2022) was used to obtain the number of orthologous genes in the meta-analysis species for the encoding nitrogen transporters [*NRT1/PTR FAMILY* (*NPF*) and *NRT2*-type for nitrate; *AMT1* and *AMT2* homologs for ammonium].

### Phylogenetic analysis of nitrogen transporters

To compare the phylogenetic resemblance of nitrogen transporters between spinach and Arabidopsis as ammonium-sensitive species as opposed to pea and rice as tolerant species, the different protein sequences were selected from TAIR (http://www.arabidopsis.org/, accessed 21 February 2022), ‘Spinach Base Genome’ (http://www.spinachbase.org/, accessed 21 February 2022), ‘Pisum sativum Cameor’ (http://www.pulsedb.org/, accessed 21 February 2022), and ‘The Rice Annotation Project’ (RAP) (https://rapdb.dna.affrc.go.jp/, accessed 22 February 2022). Then, sequences were aligned using MUSCLE (Multiple Sequence Comparison by Log-Expectation) due to its high accuracy and ability to generate precise alignments, even for highly divergent sequences as is the case with the different plant species mentioned. The selected parameters for MUSCLE were as follows: Gap open: -2.90; Gap extend: 0.00; Hydrophobicity multiplier: 1.20; Max iterations: 10 000; Cluster method: Unweighted Pair Group Method with Arithmetic Mean—UPGMA; Min. diag. Length: 24.

Finally, the phylogenetic tree was constructed by using the Maximum Likelihood method and JTT matrix-based model (1000 bootstrap iterations) in MEGA 11 software (Molecular Evolutionary Genetics Analysis, https://www.megasoftware.net/).

### Plant material and growth conditions

Spinach (*Spinacia oleracea* L. ‘Winter-Giant’) seeds were sown in vermiculite/perlite (2/1) and irrigated with distilled water. Ten days later, seedlings were transplanted to a continuously aerated hydroponic culture system and the nutrient solution was renewed every 3 d. Nitrogen-free nutrient solution was prepared: 1.15 mM K_2_HPO_4_; 2.68 mM KCl; 0.7 mM CaSO_4_; 0.07 mM Na_2_Fe-EDTA; 0.85 mM MgSO_4_; 16.5 μM Na_2_MoO_4_; 3.7 μM FeCl_3_; 3.4 μM ZnSO_4_; 16 μM H_3_BO_3_; 0.5 μM MnSO_4_; 0.1 μM CuSO_4_; 0.2 μM AlCl_3_; 0.1 μM NiCl_2_; 0.06 μM KI. The nitrogen source for the initial ammonium sensitivity study was ammonium sulfate (NH_4_)_2_SO_4_, and it was used at 1.25, 2.50, 3.75, and 5 mM NH_4_^+^. Then, to determine the effect of ammonium and nitrate co-provision on plant growth, the following ammonium to nitrate proportions were used: 0.5 mM of only nitrate (sole NO_3_^–^ nutrition), 3:4 (3.75 mM NO_3_^–^+1.25 mM NH_4_^+^), 1:1 (2.5 mM NO_3_^–^+2.5 mM NH_4_^+^), 4:3 (1.25 mM NO_3_^–^+3.75 mM NH_4_^+^), and 5 mM of only ammonium (sole NH_4_^+^ nutrition). Ca(NO_3_)_2_ and (NH_4_)_2_SO_4_ were used as nitrate and ammonium sources, respectively. The nutrient solution pH was maintained constant at around pH 6 (±0.4) by using a H_2_SO_4_ solution at a 5% concentration (~0.9 M), or at around pH 8 (±0.2) by using an NaOH solution at 5 M. In all the treatments, sulfate was chosen as a compensating ion since it is known to have relatively little effect on the uptake of other ions (Lasa *et al.*, 2001). Plants were grown under controlled conditions throughout the experiment: temperature 22/18 °C day/night, 70–60% relative humidity, and a photoperiod of 12 h at 350 µmol m^–2^ s^–1^.

Treatments lasted for 3 weeks, during which plants reached phenological stage 13–15 according to Feller *et al*. (1995).

Pea (*Pisum sativum* L. ‘Sugar-Snap’) seeds were surface-sterilized in 1% (v/v) hypochlorite, 0.01% (w/v) SDS for 40 min, rinsed with sterile water, incubated in 0.01 M HCl for 10 min, and then rinsed with sterile water again. Then, seeds were germinated at 26 °C in vermiculite/perlite (2/1) for 96 h under dark conditions. Ten-day-old seedlings were transplanted into the hydroponic culture condition, following the aforementioned conditions for spinach plants, but with a double nitrogen dose (i.e. 10 mM) due to the different sensitivity of these species to ammonium nutrition in the sole ammonium treatment: spinach at 10 mM ammonium hardly grew and it was difficult to obtain sufficient biomass for analysis, while pea at 5 mM showed no appreciable symptoms of ammonium toxicity (data not shown). Thus, 10 mM and 5 mM total nitrogen for spinach and pea were considered high doses when ammonium was the only source, and compatible with stressful physiological studies of ammonium toxicity. Pea plants were allowed to grow for 2 weeks, the time at which they reach phenological stage 13–15 (Feller *et al.*, 1995).

### Tissue soluble ion content

A 0.2 g aliquot of root or shoot material was extracted by centrifugation (15 000 g for 20 min at room temperature) from the frozen tissue to which, previously, 1 ml of autoclaved distilled water was added, and incubated in a water bath at 80 °C for 5 min. The supernatants were diluted 12.5 times with distilled water and analyzed by ion chromatography, in a 940 Professional IC Vario 2, Metrohm equipped with a conductivity detector and Metrosep C6, 150/4.0, Metroh column (cations) and Metrosep A Supp7, 150/4.0, Metrohm column (anions). A specific isocratic gradient method was used for cations with 1.7 mM nitric acid+1.7 mM dipicolinic acid as eluent with flux of 0.9 ml min^–1^, and another for anions with sodium 3.6 mM carbonate as eluent with flux of 0.9 ml min^–1^. The detected ions were the cations ammonium (NH_4_^+^), calcium (Ca^2+^), magnesium (Mg^2+^), potassium (K^+^), and sodium (Na^+^), and the anions nitrate (NO_3_^–^), nitrite (NO_2_^–^), sulfate (SO_4_^2–^), phosphate (PO_4_^3–^), and chloride (Cl^–^).

### Isotopic labeling analysis

#### ^15^Nitrogen (^15^N) uptake and translocation

For ^15^N-labeled ammonium ([^15^N]ammonium) and nitrate ([^15^N]nitrate) uptake measurements, five plants from each treatment were incubated in a sealed 50 ml Erlenmeyer flask, such that the roots were fully immersed in 50 ml of solution. [^15^N]ammonium or [^15^N]nitrate was added (≥98 atom % ^15^N as NH_4_Cl or KNO_3_, respectively) to a final concentration of 5 mM or 10 mM and at pH 6 and 8, and incubated for the indicated times: 3, 7, 15, and 30 min. Roots and shoots were harvested separately and dried in an oven at 75–80°C to a constant weight (48–72 h). Powdered and encapsulated dry plant material from each sample (shoots and roots) was separately packed in tin capsules. Total nitrogen and ^15^N-labeled nitrogen were determined by an elemental analyzer coupled to isotope ratio mass spectrometry (IRMS; Carlo Erba 1108 CHNS-O elemental analyzer coupled in continuous flow mode to an IRMS VG Isochrom isotope ratio mass spectrometer).

In addition, the percentage of [^15^N]nitrogen translocation from root to shoot for each time point was calculated based on the partition of ^15^N between shoots and total plant, as follows:


ShootN15Shoot N15+RootN15= (%N15t shoot- % N15t0 shoot)× % Nshoot×gDWshoot[( % 15Nt root- % 15Nt0 root)× % Nroot×g DWroot]+ [( % 15Ntshoot- % 15Nt0 shoot)× % Nshoot×gDWshoot] 

with %^15^N_t shoot_=percentage of ^15^N with respect to percentage total N in shoot at a certain time; %^15^N_t0 shoot=_percentage of ^15^N with respect to percentage total N in shoot at zero time; %N _shoot=_percentage total N in shoot; g DW_shoot_=biomass in shoot expressed as dry weight; %^15^N_t root_=percentage of ^15^N with respect to percentage total N in root at a certain time; %^15^N_t0 root=_percentage of ^15^N with respect to percentage total N in root at zero time; %N _root=_percentage total N in root; and g DW_root=_biomass in shoot expressed as dry weight.

The ^15^N contents (total ^15^N taken up) were obtained using δ^15^N and the total percentage of nitrogen for each plant tissue (shoots and roots) according to Ariz *et al.* (2011).

### Phytohormone analysis

Plants were harvested and separated into root and shoot before freezing in liquid nitrogen. Samples were reduced to a powder and stored at –80 °C before analyses. Quantification and data processing of phytohormones was performed following the methodology of Silva-Navas *et al.* (2019).

The content of indole acetic acid (IAA), abscisic acid (ABA), salicylic acid (SA), jasmonic acid (JA), and JA-Ile in plant tissues was analyzed by HPLC-electrospray-high-resolution accurate MS (HPLC-ESI-HRMS). These hormones were extracted and purified from 0.25 g of ground frozen plant tissue, and homogenized with 2.5 ml of pre-cooled (−20 °C) methanol:water:formic acid (90:9:1, v/v/v, with 2.5 mM Na-diethyldithiocarbamate) and 25 μl of a stock solution of 1000 ng ml^–1^ of deuterium-labeled internal standards in methanol. Samples were shaken in a ‘Multi Reax’ shaker at room temperature for 60 min at 500 g. Immediately afterward, solids were separated by centrifugation at 16 000 *g* for 10 min and re-extracted with 1.25 ml of fresh extraction mixture by shaking for 20 min and subsequent centrifugation. Aliquots of 2 ml of the pooled supernatants were separated and evaporated in a ‘RapidVap Evaporator’ operating at 40 °C. The residue was re-dissolved in 500 μl of methanol/0.133% acetic acid (40:60, v/v) and centrifuged at 20 000 *g* for 10 min before injection into the HPLC-ESI-HRMS system.

The plant endogenous content of the following cytokinins (CKs) was also analyzed: *trans*- and *cis*-zeatin (*t*Z and *c*Z), dihydrozeatin (DHZ), *trans*- and *cis*-zeatin riboside (*t*ZR and *c*ZR), dihydrozeatin riboside (DHZR), isopentenyladenine (iP), and isopentenyladenosine (iPR). The extraction process was carried out following the method described by Silva-Navas *et al.* (2019), using 0.25 g of frozen plant material previously ground with liquid nitrogen. Sample homogenization was carried out with 4 ml of pre-cooled (−20 °C) methanol:water:formic acid (15:4:1, v/v/v), and with 25 μl of a stock solution of 100 ng ml^–1^ of each deuterium-labeled standard (in methanol). An overnight extraction at −20 °C was performed, after which solids were separated (16 000 g, 10 min, 4 °C). Then, they were re-extracted with 2 ml of extraction mixture and centrifuged again (16 000 g, 10 min, 4 °C). Supernatants were passed through a Sep-Pak C18 cartridge pre-conditioned with 2 ml of methanol and 2 ml of extraction medium. Subsequently, the eluate was evaporated near to dryness with a RapidVap Evaporator, and the residue was re-dissolved in 2 ml of 1 M formic acid. This solution was applied to an Oasis MCX column pre-conditioned with 2 ml of methanol and 2 ml of 1 M formic acid. The column was washed with 2 ml of 1 M formic acid, 2 ml of methanol, and 2 ml of 0.35 M NH_4_OH, applied in succession. Finally, CK bases and ribosides were eluted with 2 ml of 0.35 M NH_4_OH in 60% methanol (v/v). The eluate was evaporated to dryness in the RapidVap Evaporator and re-dissolved with 250 μl of methanol and 250 μl of 0.04% formic acid, and centrifuged (20 000 *g* and 10 min) before injection into the HPLC-ESI-HRMS system.

### Methylglyoxal

Approximately 0.2 g of root or shoot fresh tissues were ground in a mortar with 200 µl of 5% perchloric acid, and the extract was centrifuged for 10 min at 18 000 *g*. A 10 mg aliquot of charcoal was added to decolorize the supernatant and neutralized by potassium carbonate. Then 20 µl of the supernatant was mixed with 260 µl of sodium dihydrogen phosphate and 20 µl of *N*-acetyl-l-cysteine (reaction initiator) and left to react for 15 min. The content of *N*-acetyl-*S*-(1-hydroxy-2-oxo-prop-1-yl) cysteine formed was recorded at 288 nm in a synergy™ HT Multi-Detection Microplate Reader (BioTek Instruments Inc., Winooski, VT, USA). Results were compared with the absorbance of a standard curve of methylglyoxal (MG; range of concentrations used in the calibration curve) at the same wavelength to extrapolate for the MG concentration in the sample.

### Statistical analysis

All statistical analysis was performed using Statistical Product and Service Solutions (SPSS, USA) for Windows, version 15.0. Comparisons of sample means were performed either by Student’s *t*-test (*P*<0.05) or by ANOVA (*P*<0.05) followed by Tukey’s post-hoc multiple comparisons tests, as indicated in the figure legends.

Principal component analysis (PCA) was performed using the prcomp function in R-software to compare the overall EIVs of N, R, and spread capacity with the root and shoot LnBR values of each plant species.

Heat-map visualization of log_2_ ratios of soluble ions was based on *Z*-score values and performed with publicly available Morpheus software (https://software.broadinstitute.org/morpheus/). Individual samples and ions were separated using hierarchical clustering (Ward’s algorithm), with the dendrogram being scaled to represent the distance between each branch (distance measure, Euclidean distance).

## Results

### The sensitivity of plants to ammonium can be assessed using a biomass-based indicator that is highly correlated with their EIVs and range of nitrogen transporters

**Fig. 2.**
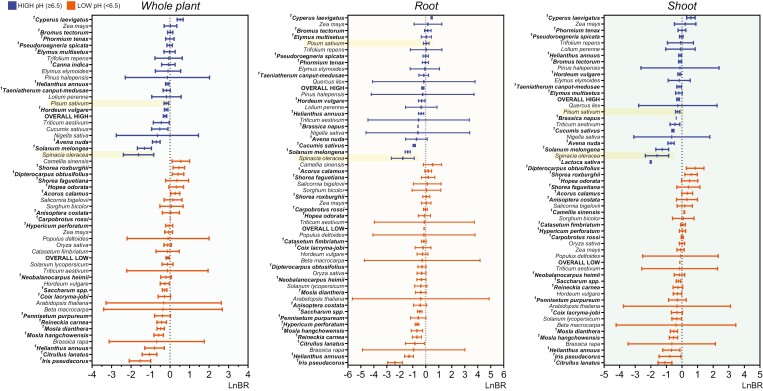
Plants’ sensitivity to ammonium can be assessed using a biomass-based indicator. Forest plots graphs depict the standardized mean effect sizes and 95% confidence intervals of sole ammonium source versus nitrate (LnBR) under high or low empirical pH (represented by symbol color) on dry weight biomass of the whole plant (A), root (B), and shoot (C) in the 50 examined plant species. An effect size of zero is indicated by a dotted line. The LnBR shows different responses in spinach and pea (highlighted in yellow). The pooled effect sizes of LnBR for the low and high pH conditions of the hydroponic nutrient solutions used in the studies are represented by OVERALL LOW and HIGH, respectively. Species in bold with superscripts were manually included in the forest plots; however, due to a shortage of studies, they were not included in the meta-analysis. See Supplementary Table S1 for reference information on the selected studies.

A systematic analysis that covered 85 cultivars, representing 50 distinct species from 16 botanical families, was performed to determine their sensitivity to ammonium as the sole source of nitrogen in hydroponic culture conditions (Fig. 1; Supplementary Table S1). In Fig. 2, the fresh biomass of the whole plant, root, and shoot were plotted separately and ordered according to the size effect of ammonium or nitrate nutrition (LnBR=natural logarithm of the ratio between the fresh biomass gained via ammonium or nitrate nutrition) at two ranges of pH: one below the optimal pH for nutrient availability and plant growth in hydroponics (i.e.<6.5) and another above or equal to this pH (pH ≥6.5; Raviv *et al.*, 2019).

The position of each species in the forest plot diagrams remained consistent for both organs (Fig. 2). This indicates that ammonium sensitivity affected both roots and shoots equally, ultimately influencing the plant’s overall biomass. Generally, species located above the overall LnBR thresholds corresponded to those traditionally known for their higher ammonium tolerance, such as *Zea mays* L. (maize), *Oryza sativa* L. (rice), or *Sorghum bicolor* L. (sorghum), in contrast to horticultural species such as *Spinacia oleracea* L. (spinach), *Lactuca sativa* L. (lettuce), or *Cucumis sativus* L. (cucumber). It is worth noting how the LnBR index responded to pH differently in certain species: *Helianthus annus* L. (sunflower) and *Hordeum vulgare* L. (barley) demonstrated increased ammonium tolerance at higher pH levels, while maize was relatively unaffected by the pH. Thus, the external pH of nutrient solutions has a distinct impact on plant biomass in ammonium nutrition compared with nitrate, which varies across plant species.

**Fig. 3.**
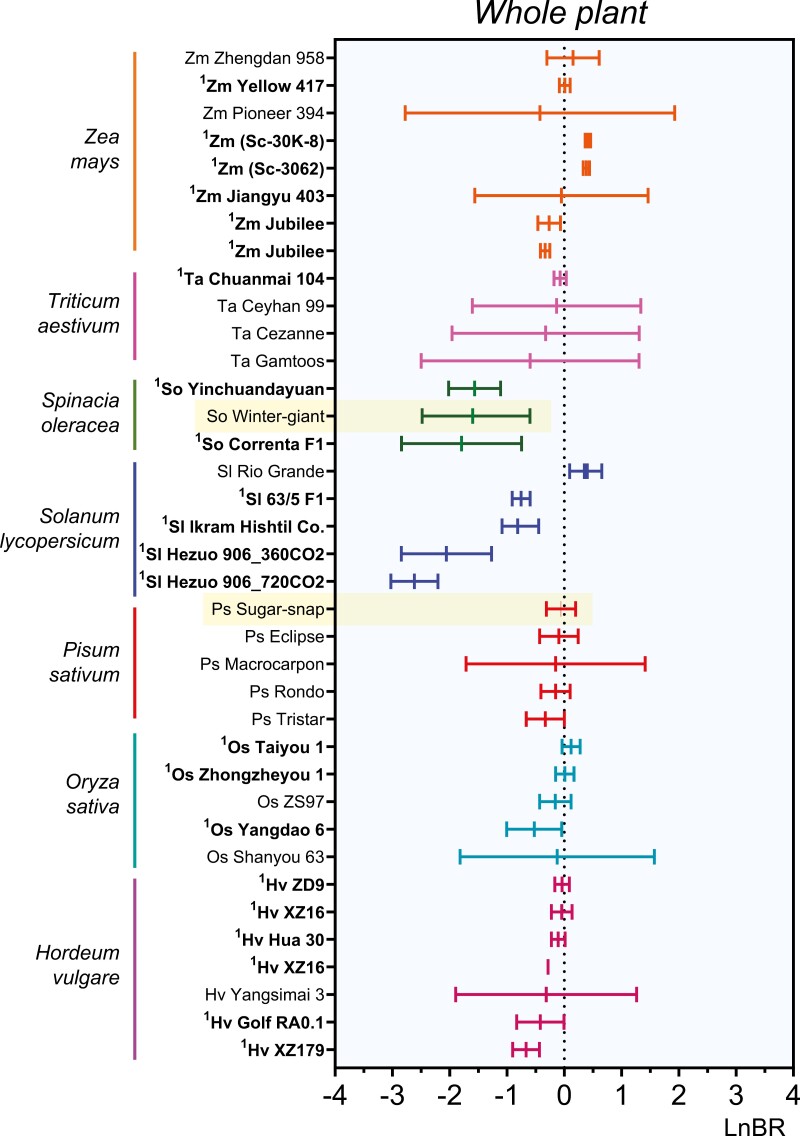
Meta-analysis of the biomass-based indicator shows intraspecific variability in the response of cultivated plant species to ammonium nutrition. The effect size in biomass production of ammonium nutrition relative to nitrate, depicted in a forest plot graph. LnBR shows the different degree of ammonium sensitivity of plant cultivars within some relevant crops included in the meta-analysis. All data are standardized mean effect sizes and the 95% confidence interval. The dotted line illustrates an effect size of zero. The specific spinach and pea cultivars used in the subsequent analytical comparison are highlighted in yellow. The forest plot included certain varieties, shown in bold with superscript, which were manually added, because they were present in only one study (and thus excluded from the meta-analysis) Supplementary Table S1 contains references of selected studies for each species.

Since some of the analyzed species included various cultivars, their intraspecific variability of LnBR was represented in Fig. 3 to illustrate potential differences in ammonium sensitivity among varieties or cultivars. Maize, barley, rice, and, in particular, *Solanum lycopersicum* L. (tomato) exhibited significant variability of LnBR among cultivars, while *Triticum aestivum* L. (wheat), pea, and spinach showed minimal variation. For subsequent analyses, the ‘Winter-Giant’ spinach (LnBR –1.71 ± 0.34) and ‘Sugar-Snap’ pea varieties (LnBR –0.45 ± 1.31) were specifically selected due to their contrasting levels of ammonium tolerance (Figs 2, 3).

**Fig. 4.**
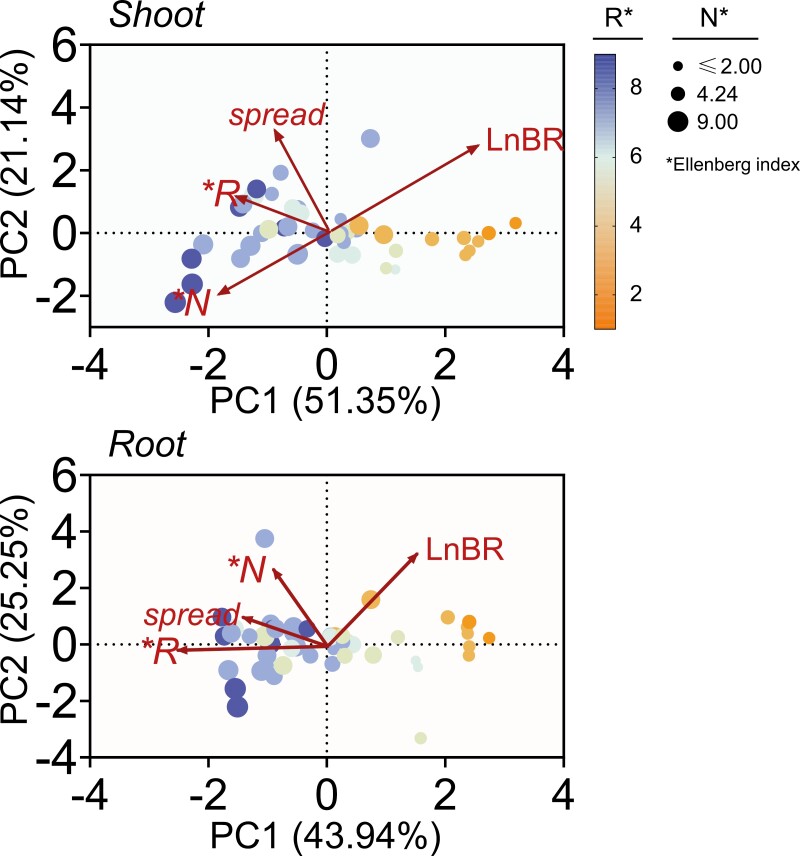
Nitrophily is the most influential ecological trait in defining the first component of the PCA. This attribute accounts for around half of the variation in data for plants’ LnBR values. Specifically, in the shoot and roots, this first component explains 51% and 44% of the variation, respectively, while the second component accounts for 21% and 25%. The soil pH (‘R’) is displayed through a color scale, while the plot size indicates fertility and nitrogen (‘N’) preferences based on the Ellenberg index.

When analyzing the LnBR values of various plant species in relation to soil fertility parameters such as the EIVs of soil pH (R) and nitrogen requirement (N), the PCA revealed two main components in both shoot and root, explaining 72.5% and 69.2% of the variability, respectively (Fig. 4). PC1 primarily reflected the ‘nitrophily’ in shoot, with species associated with acidic soils and low fertility/nitrogen requirement positioned to the right. Conversely, plant species associated with neutral and alkaline soils and higher N values were clustered in the lower left quadrant of PC1–PC2. LnBR values in the shoot were inversely correlated with N values, suggesting that species with higher ammonium tolerance require less fertile or nitrogen-rich soil. Fertility/nitrogen requirement in the root correlated closely with spread capacity and, to a lesser extent, soil pH.

**Fig. 5.**
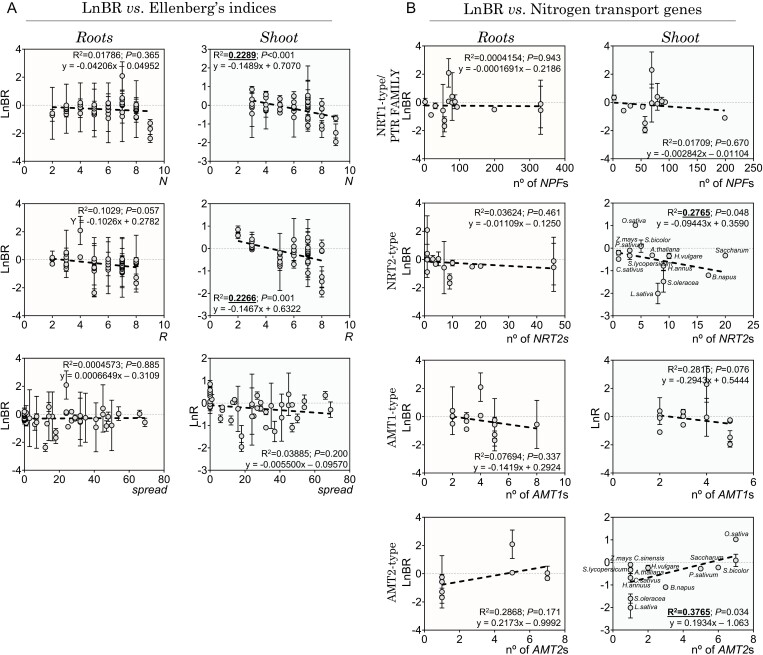
LnBR values correlate with soil pH, nitrophily, and the number of genes coding for *NRT2*- and *AMT2*-type transporters. There is a significant correlation between the ammonium sensitivity indicator (LnBR) and some Ellenberg’s indices (A), as well as the number of nitrogen transporter genes present in the plant species examined (B). The presented data show the mean LnBR ±SD adjusted to linear correlation (*R*^2^ and *P*). The statistically significant correlation between LnR values and the number of *NRT2*- and *AMT2*-type genes is highlighted in bold. N indicates nitrophility/soil fertility and R indicates soil pH according to the Ellenberg indices. Additionally, the number of countries where the species can be invasive is reported as ‘spread’.

Comparison of LnBR values between species exhibited a significant negative correlation in the shoot with N (*R*^2^=0.23, *P*<0.001) and R values (*R*^2^=0.23, *P=*0.001) (Fig. 5A), indicating that plants adapted to acidic soils and with lower nitrogen requirements show higher tolerance to ammonium nutrition. Acidophilic species tended to group closely together, reflecting specific habitat preferences, while species adapted to neutral and alkaline soils exhibited a wider range along the PC2 axis (Fig. 4).

A targeted *in silico* analysis of the nitrogen transporters repertoire, based on available plant genomes or orthology with *Arabidopsis thaliana* L. (Arabidopsis), allowed comparison of the number of nitrogen transport genes in only 14 of the 50 plant species studied (Supplementary Table S2; Supplementary Figs S1–S3). A significant negative correlation was found between the number of high-affinity nitrate genes (i.e. those encoding NRT2-type proteins) and shoot LnBR values (*R*^2^=0.28, *P*=0.048; Fig. 5B; Supplementary Table S2; Supplementary Fig. S2). Shoot LnBR values, indicating greater sensitivity to ammonium, were notably negative in spinach and *Brassica napus* L. (rapeseed). These two plant species showed the highest number of *NRT2* orthologous members compared with Arabidopsis, with spinach having nine, rapeseed 17, and Arabidopsis seven *NRT2* members (Fig. 5B; Supplementary Table S2). However, plant species that exhibit greater tolerance to ammonium, such as rice and sorghum, have four and five members of *NRT2*, respectively, or a single member in the case of pea, with a wider range of tolerance to ammonium (Fig. 5B; Supplementary Table S2).

Regarding ammonium transporters, a negative correlation was observed in shoots between LnBR values and *AMT1*-type genes, while a positive association was found for *AMT2*-type homologs (*R*^2^=0.38, *P*=0.034; Fig. 5B). Most of the species analyzed were found to be more sensitive to ammonium, based on the negative LnBR values. This was particularly true for species with <4 *AMT2* homologs. Plant species showing greater tolerance were on the opposite side of the spectrum, such as rice and sorghum, with seven *AMT2* homologs, *Saccharum officinarum* L. (sugarcane) with six, or pea with five *AMT2*-type homologs (see Supplementary Table S2; Supplementary Fig. S3).

It is noteworthy that the correlation between the number of nitrogen transport genes, and N and R EIVs, with ammonium sensitivity is consistently stronger in shoots than in roots (see Figs 4 and 5A). This could be attributed to the heightened susceptibility of the shoot to ammonium-dependent acidic disturbance (Hachiya *et al.*, 2021).

### Testing of plant growth and nitrogen uptake capacity in two plant species with divergent positions on the ammonium sensitivity ‘scale’: spinach versus pea

Out of the 50 analyzed plant species, at least 19 were recognized as cultivated species. Following an examination of the results from Figs 2 and 3, two cultivated plant species exhibiting divergent responses on the ammonium tolerance ‘scale’ were selected for further investigations: spinach cv. ‘Winter-Giant’ and pea cv. ‘Sugar-Snap’. Subsequent experiments delved into multiple developmental, nutritional, and metabolic indicators to comprehensively evaluate their responses to nitrogen nutrition and pH.

#### Spinach and pea plants exhibited contrasting nutritional and developmental responses to nitrogen form and pH

**Fig. 6.**
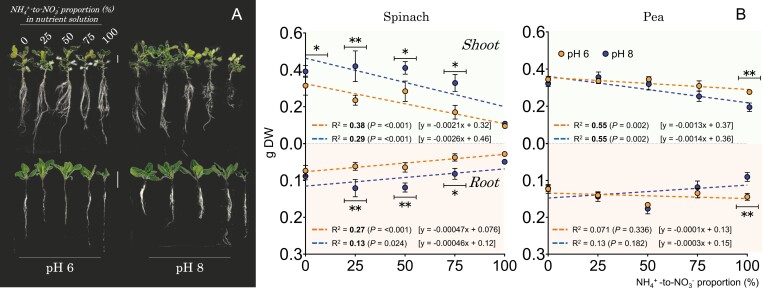
Nitrate in co-provision with ammonium promotes growth in both spinach, which shows a boost at pH 8, and pea, which is unaffected by pH levels. (A) The phenotype of hydroponically cultivated spinach and pea plants, with white vertical lines indicating a scale of 5 cm. (B) The connection between biomass of the shoot and root, the proportion of ammonium to nitrate, and pH levels. As the proportion of ammonium to nitrate in the nutrient solution increases, biomass decreases. The trend is quantified by *R*^2^ and is represented by the dashed lines. Statistical significance is shown in bold, with a significance level of *P*≤0.05. Asterisks indicate significant differences in pH as determined by Student *t*-test. *0.05>*P*>0.01, **0.01>*P*>0.001. Spinach and pea plants were grown for 3 weeks and 2 weeks, respectively, and were exposed to varying proportions of ammonium and nitrate, ranging from sole nitrate to sole ammonium continuously. The total nitrogen level was 5 mM for spinach and 10 mM for pea plants.

The detrimental impact of exclusive ammonium nutrition on plant growth was confirmed in both plant species. Spinach growth was significantly reduced under exclusive ammonium conditions, irrespective of pH and ammonium concentration (i.e. showing similar ‘poor’ growth from 1.25 mM to 5 mM ammonium as the sole nitrogen source; Fig. 6; Supplementary Fig. S4). Pea plants were affected by ammonium nutrition in a concentration- and pH-dependent manner, with notable growth impairment observed at higher concentrations of ammonium (i.e. 10 mM) and pH 8 (Fig. 6; Supplementary Fig. S4).

The provision of nitrate even at its lower proportion compared with ammonium (i.e. 1.25 mM compared with 3.75 mM ammonium) not only largely alleviated the ammonium toxicity but also enhanced biomass production in spinach plants (Fig. 6A, B). Maximum shoot biomass in spinach was achieved at an ammonium to nitrate proportion of ≤50% and at pH 8 (Fig. 6B). These findings emphasize the pivotal role of pH in promoting nitrate-mediated growth in spinach plants, while sole ammonium nutrition was toxic for spinach regardless of pH (as illustrated in Supplementary Fig. S4). Nitrate and external pH also influenced pea growth; however, unlike spinach, the beneficial effect of nitrate on pea root and shoot biomass was not dependent on the pH. Additionally, in pea, the influence of pH was only noticeable in the absence of nitrate, leading to stimulated plant growth at pH 6 (Fig. 6B).

**Fig. 7.**
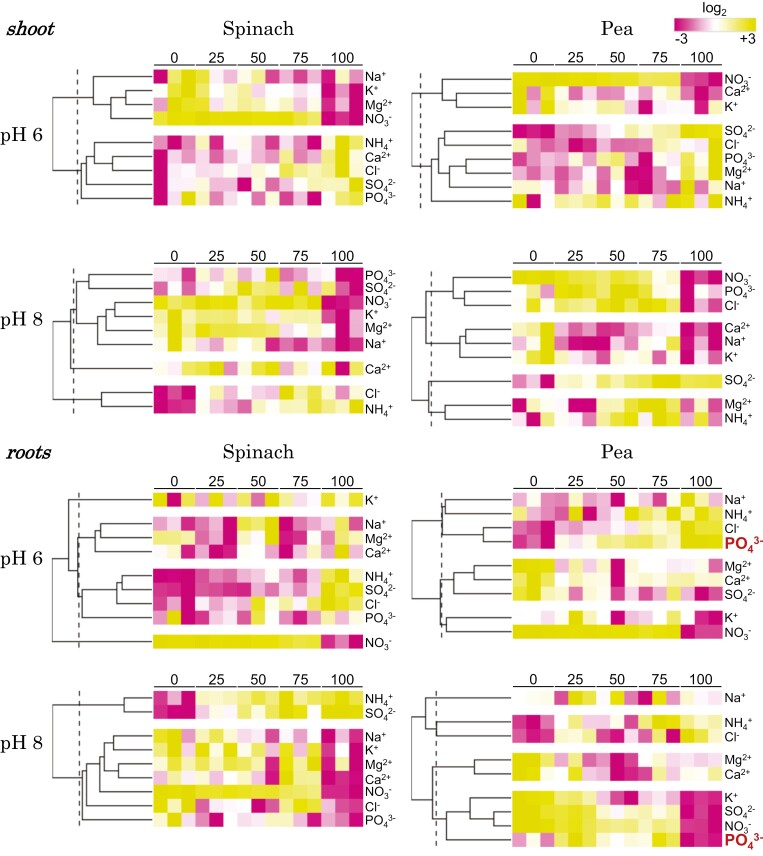
Mineral nutrition status was analyzed through a heatmap, utilizing *Z*-score values of average log_2_ soluble ions in replicates from both the shoot and root of spinach and pea plants at pH 6 and 8. The rows of the heatmap represent ions, and the columns display the percentage ratio of ammonium to nitrate (with 0 indicating only nitrate and 100 indicating only ammonium). Ions with significantly lower levels are highlighted in purple, while those with significantly higher levels are shown in yellow. The brightness of each color corresponds to the magnitude of the difference when compared with the average value. Individual samples and ions are separated using hierarchical clustering utilizing Ward’s algorithm. The dendrogram is scaled to represent the distance between each branch through a distance measure of Euclidean distance. Highlighted in red are the greatest differences found between pHs, which were the high and low phosphate contents in pea roots at pH 6 and 8, respectively. This visualization was produced utilizing the publicly available Morpheus software (Broad Institute). A total of 5 mM nitrogen was applied for spinach and 10 mM for pea plants.

The ion content of plant tissues was studied because the availability of other nutrients is also dependent on external pH. While the tissue ion content of spinach did not explain its superior growth under pH 8 conditions, it was effective for pea plants under pH 6 conditions (Fig. 7). Roots of both species accumulated ammonium ions, especially at pH 8, concomitant with the ammonium to nitrate proportion in the nutrient solution. This trend was consistent in spinach shoots regardless of pH, with ammonium content exceeding that of pea shoots, even though spinach was grown at half the total nitrogen concentration (Supplementary Fig. S5). Nitrate accumulation in roots and shoots was similarly favored at pH 6 in both species, as shown in Fig. 7 and Supplementary Fig. S5, highlighting the higher nitrate uptake capacity in response to reduced nitrate availability at lower pH (Marschner, 2012). Despite spinach being cultivated with only half the total nitrogen supplied to pea plants, the slight difference in nitrate content confirms the remarkable capacity of spinach to accumulate nitrate compared with pea plants (Supplementary Fig. S5).

For the other soluble ions, there were no significant depletions in cations (Supplementary Fig. S6) and anions (Supplementary Fig. S7) in spinach tissues that could explain the lower growth at pH 6. The heatmap (Fig. 7) revealed a general tendency towards a more pronounced reduction in the main competitive cations with ammonium (K^+^ and Mg^2+^) in spinach compared with pea as the proportion of external ammonium to nitrate increased, irrespective of pH. Interestingly, pea plants accumulated higher levels of SO_4_^2–^ and PO_4_^3–^ in shoot and root at pH 6 with an increasing proportion of ammonium to nitrate. Conversely, spinach plants exhibited minimal changes in response to nitrogen and pH treatments (Fig. 7; Supplementary Fig. S7).

#### Spinach and pea plants showed contrasting capacities for nitrate and ammonium uptake and translocation in response to nitrogen forms and pH

Since the distinct sensitivity to ammonium nutrition appears to depend on the preference for nitrate (e.g. higher in spinach) and other nutrients whose availability depends in turn on the pH (e.g. phosphorus in pea), a ^15^N uptake assay was conducted to assess their uptake capacity to acquire nitrate and ammonium.

**Fig. 8.**
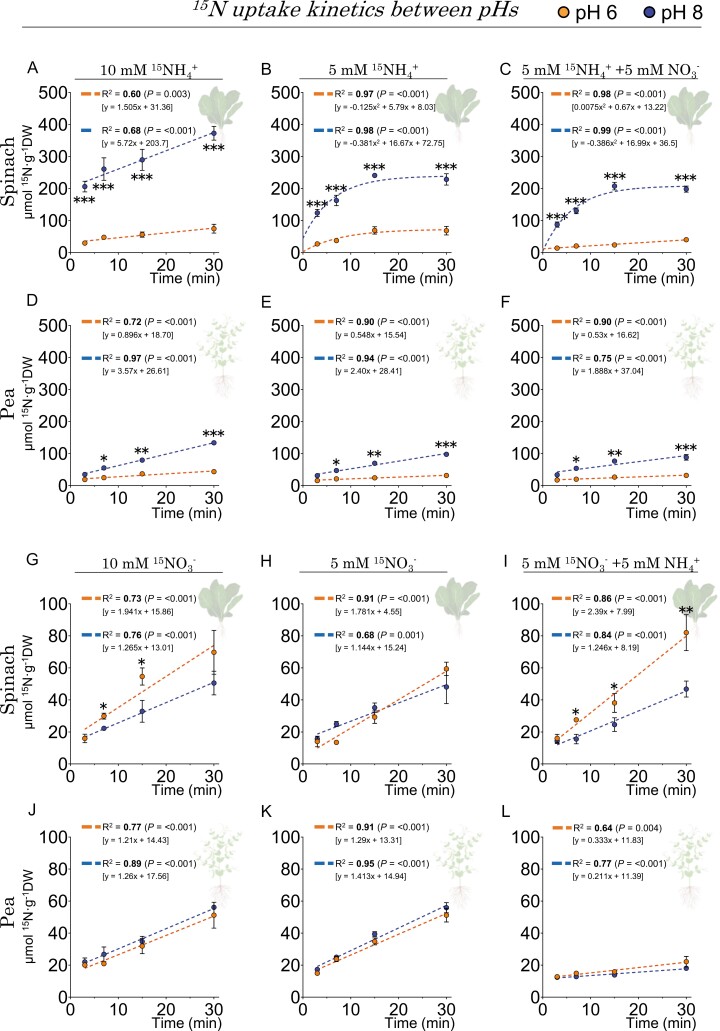
Mutual modulation of nitrate and ammonium uptake occurred in spinach and pea plants when both nitrogen sources were co-supplied, with dependence on pH. ^15^N influx was recorded following a 30 min incubation of ^15^N-labeled concentrations of NH_4_^+^ (A–F) or NO_3_^–^ (G–L) at levels of 5 mM and 10 mM, at pH 6 and 8. Data represent the means ±SE (*n*=3–5). The dashed lines indicate the trend in the ^15^N content during incubation, measured by *R*^2^, with statistical significance indicated in bold (*P*≤0.05). Asterisks represent differences between pHs at each time point (Student *t*-test). *0.05>*P*>0.01, **0.01>*P*>0.001; ***<0.001. The direct comparison of ^15^N influx between species is shown in Supplementary Fig. S8.

The results indicate that there was a significant increase in the uptake of [^15^N]ammonium at pH 8 compared with pH 6 (Fig. 8A–F). In particular, spinach exhibited a more significant difference as compared with pea (Supplementary Fig. S8A–F). When both nitrogen sources were available at pH 8, >80% of nitrogen uptake in spinach was attributable to [^15^N]ammonium, whereas at pH 6 it accounted for only ~25% (Fig. 8C, I). At pH 8, pea plants exhibit a [^15^N]ammonium uptake rate of 80% of total nitrogen uptake, while at pH 6 it represented ~40% (Fig. 8F, L).

**Fig. 9.**
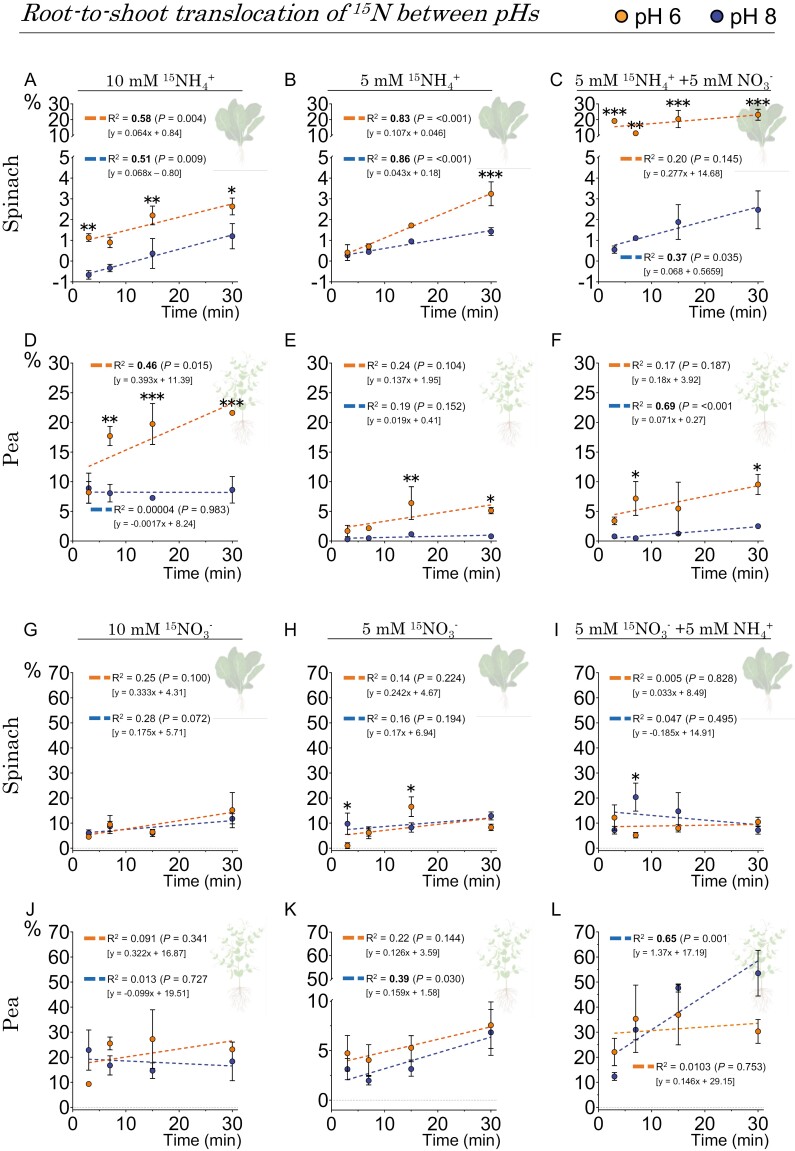
The translocation of nitrate and ammonium from the roots to the shoots in spinach and pea plants is influenced by their co-provision and pH levels. The percentage of translocated ^15^N-labeled NH_4_^+^ (A–F) or ^15^N-labeled NO_3_^–^ (G–L) in the shoots was measured after a 30 min incubation at concentrations of 5 mM and 10 mM and pH values of 6 and 8. Data represent the means ±SE (*n*=3–5). The dashed lines indicate the trend in the ^15^N content in the shoot with respect to the total ^15^N in the plant over the incubation period. *R*^2^ quantifies this trend, and its statistical significance is indicated in bold (*P*≤0.05). pH differences at each time point are denoted by asterisks based on the Student *t*-test. Significance levels are indicated by *0.05>*P*>0.01, **0.01>*P*>0.001, and ***<0.001. The direct contrast of ^15^N translocation between species is available in Supplementary Fig. S9.

Moreover, the percentage of root-to-shoot ^15^N translocation derived from ammonium was consistently higher at pH 6 than at pH 8, as shown in Fig. 9A–F. Additionally, increased ammonium concentrations (10 mM) in pea plants led to greater root-to-shoot translocation, as shown in Fig. 9D and E and Supplementary Fig. S9A–F.

At this stage, there are still two essential questions to address.

##### (i) How did nitrate impact ammonium uptake?

**Fig. 10.**
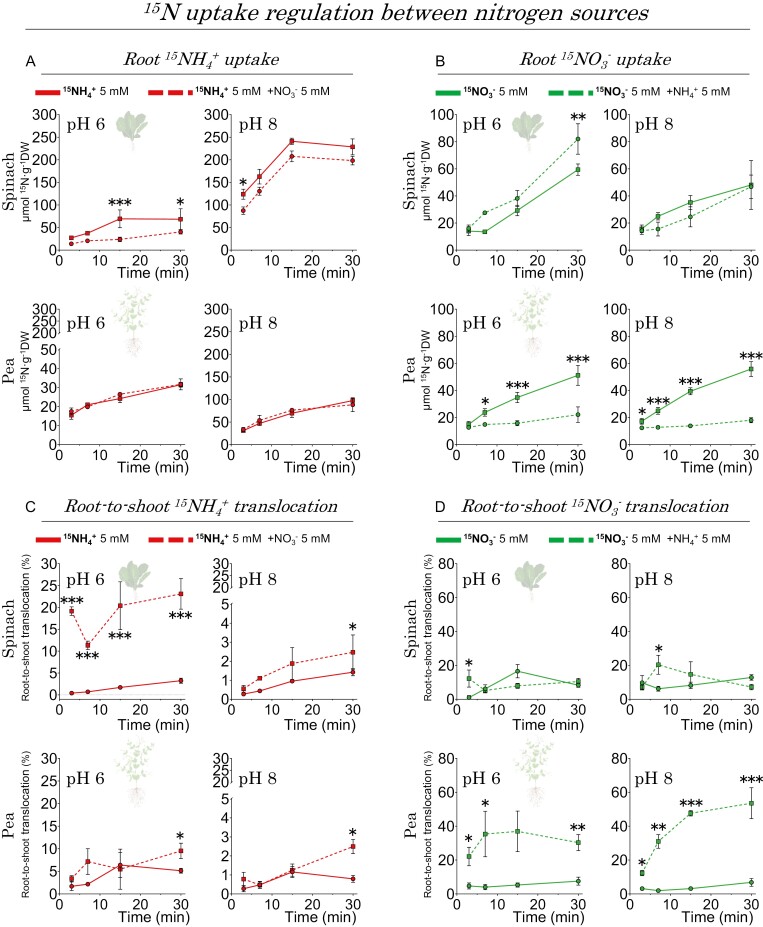
Differential transport patterns of inorganic nitrogen forms in spinach and pea, under co-provision of both nitrogen sources. The study measured 5 mM ^15^N-labeled NH_4_^+^ influx (A) and its root to shoot translocation (C), with and without 5 mM nitrate; and 5 mM ^15^N-labeled NO_3_^–^ influx (B) and its root to shoot translocation (D), with and without 5 mM ammonium. The data represent the means ±SE (*n*=3–5). The dashed lines indicate the trend in ^15^N content in plants (A and B) or percentage of translocated labeled N (C and D) over the incubation period. These trends were quantified by *R*^2^, and statistical significance is denoted in bold (*P*≤0.05). Single and co-provisioned nitrogen sources are compared using Student *t*-tests, with asterisks indicating significant differences; specifically, *0.05>*P*>0.01, **0.01>*P*>0.001, and ***< 0.001.

In spinach, the presence of 5 mM nitrate substantially decreased the uptake of [^15^N]ammonium at the two tested pH levels (Fig. 10A). Conversely, in the case of pea, nitrate did not modify the uptake of [^15^N]ammonium at the same ammonium concentration (Fig. 10A). These findings indicate that nitrate has a distinct impact on ammonium uptake in these two species, acting as an inhibitor for spinach but not for pea.

##### (ii) How did ammonium affect nitrate uptake?

In spinach, unlike in pea plants, ammonium did not decrease [^15^N]nitrate uptake; in fact, at pH 6, it even exhibited a significant increase (Fig. 10B). Spinach, categorized as a ammonium-sensitive species, appeared to have a greater ability to acquire nitrate even in the presence of high ammonium levels, failing to respond to the ‘satiety signal’ for nitrogen as reported by Okamoto *et al*. (2019).

In addition, when both nitrogen sources were provided together, opposite effects on the translocation of nitrogen derived from the two forms from roots to shoots were observed. Co-supply of nitrate and ammonium resulted in a significant increase in the translocation of ammonium-derived ^15^N in spinach, especially at pH 6 (Fig. 10C). Conversely, in pea, there was a significant increase in translocation of ^15^N derived from nitrate (Fig. 10D).

### In spinach and pea plants, can the hormonal profile or other indicators of metabolic disruption be used as indicators of ammonium toxicity?

**Fig. 11.**
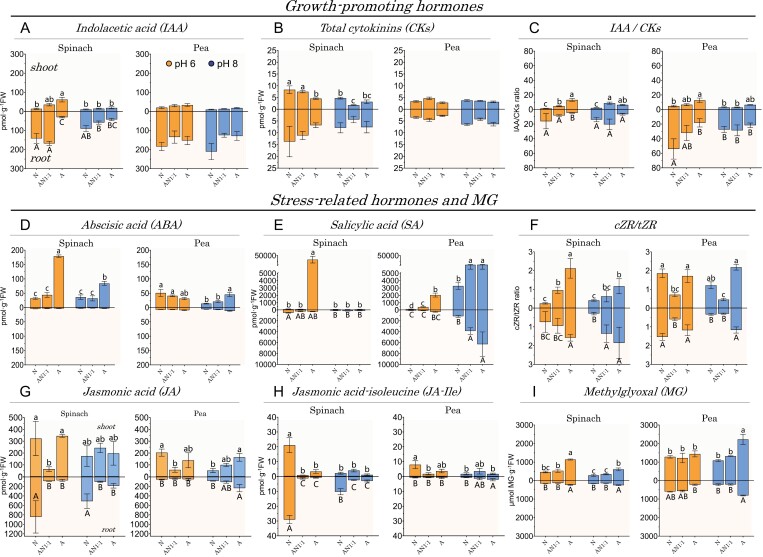
The combined use of nitrate and ammonium induces varied effects on growth-promoting hormones as well as hormones and metabolites associated with stress response, depending on the pH and species involved. The content and ratio of growth-promoting hormones (A–C), hormones related to stress response (D–H), and methylglyoxal (I) were measured in the shoot and root of spinach and pea plants treated with nitrate (‘N’), ammonium (‘A’), or equimolar co-provisions of ammonium and nitrate (‘AN1:1’) at pH 6 and 8. The data represent means ±SE (*n*=3–5). Different letters indicate significant differences according to Tukey’s test (*P*<0.05) between treatments. Spinach and pea plants were grown for 3 and 2 weeks, respectively, with a continuous supply of nitrate [Ca(NO_3_)_2_], ammonium [(NH_4_)_2_SO_4_], or equimolar co-provision of both [Ca(NO_3_)_2_+(NH_4_)_2_SO_4_]. A total of 5 mM nitrogen was applied for spinach and 10 mM for pea plants.

Hormone levels, such as of IAA of CKs, and the IAA to CK ratio, as an indicator of CK-dependent regulation of IAA biosynthesis, and vice versa (Jones *et al.*, 2010), were evaluated in terms of their function as metabolic markers for the regulation of metabolism and potential disruptions. In this regard, nitrate, but not ammonium, increased the growth-promoting CKs in shoots and the IAA to CK ratio in roots for both species (Fig. 11C). Furthermore, while pH and nitrogen treatments did not affect IAA and total CKs levels in pea plants, in spinach, nitrate stimulated accumulation of IAA and CKs in roots and CKs in shoots at pH 6 (Fig. 11A, B).

Due to the stressful situations caused by ammonium, the degree of ammonium sensitivity appears to be related to some adaptive characteristics related to pH and the capacity for nitrate and ammonium uptake, as previously shown. Analyzing certain metabolic indicators of disturbance, such as the *c*ZR to *t*ZR ratio, and contents of ABA, SA, JAs, and MG (Borysiuk *et al.*, 2018), could be useful to confirm this connection.

When the ratio of *cis*- and *trans*-zeatin ribosides was calculated (non-riboside were below the detection limit, i.e. *c*Z and *t*Z), a higher proportion of *c*ZR/*t*ZR was observed in sole ammonium-fed plants of both species (Fig. 11F). Furthermore, at the less suitable pH level for each plant species—pH 6 for spinach and pH 8 for pea—the differences from the sole ammonium treatment were even more significant (Fig. 11F). In addition, sole ammonium nutrition produced the highest contents of ABA, SA, and MG, especially in spinach at pH 6, and in pea at pH 8 (Fig. 11D, E), reflecting that the degree of sensitivity to ammonium is highly dependent on the pH. Thus, indicators of metabolic disturbance patterns support a pH-dependent sensitivity to ammonium in spinach and pea plants. In contrast, JA and its biologically active component JA-Ile showed a tendency to accumulate significantly more in spinach root and shoot under sole nitrate nutrition at pH 6 (Fig. 11G, H).

## Discussion

### The preference for nitrogen form defines plant sensitivity to ammonium in a pH-dependent manner

The degree of sensitivity to ammonium varies significantly among different plant species and even among cultivars within the same species. The observed negative correlations of N and R with shoot LnBR values indicate that species adapted to more acidic environments, such as rice (Wang *et al.*, 1993), sorghum (Feng *et al.*, 1994), or certain pea cultivars (Pintar *et al.*, 2021), demonstrate higher tolerance to ammonium (Figs 2–5). Conversely, the inverse relationship displayed by the spread capacity indicator versus shoot LnBR values implies that the capacity of plant species to thrive in diverse habitats is linked to their elevated fertility/nitrogen demands and an adaptation to a broader pH range. This dichotomy aligns with the prevalence of nitrate-preferential species in agricultural and urban settings, contrasting with forests and remote wilderness areas (Marschner, 2012).

A targeted *in silico* analysis of nitrogen transporter gene repertoires suggests that plant species with an expanded array of genes encoding high-affinity nitrogen transporters, particularly NRT2-type and, to a lesser extent, AMT1-type, appear to exhibit a tendency towards increased sensitivity to ammonium toxicity. For instance, this pattern is observed in species highly sensitive to ammonium, such as rapeseed with 17 genes encoding NRT2-type proteins, spinach with nine genes, and lettuce with eight genes. Conversely, plant species harboring a higher number of AMT2-type homologs, such as rice, sorghum, or sugarcane, seem to be associated with their reported enhanced tolerance to ammonium toxicity (Fig. 5).

The crucial role of high-affinity NRT2-type transporters in nitrate acquisition under nitrogen-limiting conditions is well established. For instance, NRT2.1 has been proposed as a nitrate sensor due to its high affinity for nitrate and its impact on root architecture under nitrogen-limiting conditions (Krouk *et al.*, 2006; Ohkubo *et al.*, 2021). Consequently, the expansion of NRT2-type transporters in nitrate-dependent/hyperaccumulator plant species such as spinach may be considered an adaptive response to cope with its scarcity in the soil, as well as low pH and ammonium toxicity (Krouk *et al.*, 2006; Hachiya *et al.*, 2011). On the other hand, the greater presence of *AMT2* homologs encoding low-affinity ammonium transporters is likely to be associated with environments characterized by higher ammonium availability, such as primary forests or flooded soils, which generally exhibit acidity and low nitrification rates (Britto and Kronzucker, 2002; Hawkesford *et al.*, 2012).

Comprehending the evolutionary adaptation within taxonomic families is key to understanding their differential response to nitrogen sources (Supplementary Figs S7–S9). For example, within the family *Leguminosae*, long-distance nitrate transport is significantly reduced compared with non-legume species, and different organic compounds may be involved in long-distance nitrogen transport according to the different clades: tropical species mainly use ureides, while temperate species use amides (Gu *et al.*, 2022). Indeed, long-distance phloem transport of organic nitrogen occurs mainly for amides in pea, whose genome does not rely on Arabidopsis orthologs of nitrate phloem transporters (NRT1.7, NRT1.9, NRT1.11, and NRT1.12; Gu *et al.*, 2022). The presence of only one *NRT2*-type gene in pea (Supplementary Table S2; Supplementary Fig. S2) may indicate a reduced dependence on nitrate, possibly compensated by the supply of ammonium from nodule nitrogen fixation, which explains the concomitant increase in the repertoire of AMT2-type proteins (Supplementary Table S2; Supplementary Fig. S3). AMT2-type transporters play a key role in the ammonium translocation from root to shoot in Arabidopsis (Giehl *et al.*, 2017), and in *Lotus japonicus* R., sorghum, and the legume *Medicago truncatula* from the arbuscular mycorrhizal symbiosis (Breuillin-Sessoms *et al.*, 2015), probably indicating a better use of ammonium with less damage to the plant. Therefore, plant species with fewer *AMT2* homologs but more *NRT2* genes, together with lower LnBR values [e.g. rapeseed, lettuce, or spinach (Fig. 3; Supplementary Table S2; Supplementary Figs S2, S3)] would be consistent with their greater nitrate dependence and ammonium sensitivity.

Growing spinach (which is characterized by low LnBR values and possesses nine *NRT2* genes along with one *AMT2* homolog) and pea (which exhibits greater plasticity in LnBR values with only one *NRT2* gene but five *AMT2* homologs) under elevated concentrations of ammonium as the exclusive nitrogen source led to a significant reduction in both root and shoot biomass (Fig. 6; Supplementary Fig. S4). However, there were notable differences in the response between the two plant species. Unlike pea, which showed amelioration of ammonium toxicity under acidic pH and low ammonium concentrations, spinach showed no relief under similar conditions, underscoring its heightened sensitivity to ammonium. Replacing part of the ammonium with nitrate rapidly alleviated the toxic effects of ammonium nutrition, irrespective of concentration and environmental pH. Co-supply of ammonium and nitrate induced a significant increase in biomass, which was particularly evident in spinach shoots, suggesting that its ammonium sensitivity can be directly related to the ‘absence of nitrate’ in such nitrate-dependent/hyperaccumulator species. It is unlikely that pH was an influencing factor in the nitrate-dependent growth promotion of pea plants, which had a detrimental effect on the availability of other important nutrients such as PO_4_^3–^, whose availability is higher at pH 6 than at pH 8 (Marschner, 2012). This may be indicative of the distinct pH-dependent availability of important macronutrients for each species, such as nitrate, K^+^, or Mg^2+^ for spinach, whose availability increases in slightly alkaline nutrient solutions, or PO_4_^3–^ and SO_4_^2–^ for pea, with higher solubility in slightly acidic media (Marschner, 2012). In pea, the growth increase in the co-provision of nitrate and ammonium was not pH dependent, but it was under sole ammonium nutrition probably due to increased PO_4_^3–^ availability, which is critical for root development (Supplementary Fig. S7C). As a higher environmental ammonium concentration increased uptake of phosphate, the principal anion maintaining charge balance against ammonium uptake (Marschner, 2012; Tian *et al.*, 2021), a lower pH and a higher ammonium to nitrate proportion could be conditions to optimize pea growth.

### Nitrate modulates ammonium transport processes in spinach but not in pea plants

The higher rates of ammonium uptake compared with nitrate in spinach and pea suggest that ammonium is absorbed more rapidly than nitrate by the roots probably due to its lower assimilation cost (Kronzucker *et al.*, 2001). The ability of ammonium to interconvert between its cationic and neutral forms in a pH-dependent manner increases its uptake capacity as the pH rises (p*K*a NH_3_=9.3; ~10% at pH 8), due to the higher abundance of the most permeable form, NH_3_ (Downing and Merkens, 1955). The fact that spinach increased ammonium uptake to a greater extent than pea at pH 8 compared with pH 6, may suggest a relationship with root plasma membrane aquaporins (Coskun *et al.*, 2013). Indeed, the spinach genome contains at least seven PIP (plasma membrane intrinsic proteins) genes compared with the five identified in pea (Song *et al.*, 2016), and a positive linear relationship between water and nitrate contents in tissues has been reported in nitrate-hyperaccumulator species (e.g. *Brassica rapa* L. ssp. *pekinensis*, Chinese cabbage; *Brassica rapa* L., field mustard; spinach; and lettuce) (Reinink *et al.*, 1987; Cárdenas-Navarro *et al.*, 1999; Burns *et al.*, 2011). Thus, it seems that a higher aquaporin-mediated hydraulic conductivity in leafy vegetables supports nitrate accumulation. However, the tonoplast intrinsic membrane proteins (the TIP family of aquaporins) have only been shown to allow NH_3_ permeation in plants (Loqué *et al.*, 2005), so the suggestion of higher NH_3_ uptake at alkaline pH mediated by aquaporins should be further investigated.

In the context of co-supply and mutual modulation between nitrate and ammonium, it is evident that nitrate exerts an inhibitory effect on ammonium uptake in spinach, but not in pea plants. So far, nitrate has been reported to have a measurable suppressive effect on ammonium uptake in *Camelia sinensis* L. (tea) (Ruan *et al.*, 2016), wheat (Du *et al.*, 2021), and rice (Yan *et al*., 2023). While the underlying cause of this suppression requires further investigation, the impact of nitrate on these species is advantageous, as it mitigates the overall ammonium influx and its associated toxicity.

On the other hand, ammonium increased net nitrate uptake at pH 6 in spinach, but decreased it in pea (Figs 8, 10). Plants that grow better with nitrate and have an increased capacity to accumulate nitrate do not become ‘saturated’ with it even when there is plenty of ammonium in the medium (Okamoto *et al.*, 2019). In other words, hyperaccumulating species would take up nitrate despite being saturated with nitrogen because they can accumulate it as an osmolyte (Burns *et al.*, 2011). Indeed, nitrate serves as an important, metabolically benign osmotic agent in such species, as it can balance other ions such as K^+^ in the plant tissues and help maintain a favorable cellular water status (Burns *et al.*, 2011). In pea, inhibition of nitrate uptake by ammonium is the most commonly reported behavior in the literature [e.g. in the pea variety ‘Marma’ (Oscarson *et al.*, 1987), *Glycine max* L. (soybean) (Chaillou *et al.*, 1994), rice (Kronzucker *et al.*, 1999), wheat (Jackson *et al.*, 1976), barley (Aslam *et al.*, 1994), sunflower (de la Haba *et al.*, 1990), maize (Volk, 1997), *Pinus pinaster* Ait. (pine) (Gobert and Plassard, 2007), and Arabidopsis (Krouk *et al.*, 2006). This uptake inhibition did not occur with the application of a glutamine synthetase (GS) inhibitor such as methionine sulfoximine (MSX), or a glutamine oxoglutarate aminotransferase (GOGAT) inhibitor such as azaserine (AZA), which prevent ammonium assimilation into glutamine and glutamate, respectively. It therefore appears that nitrate uptake is somehow regulated by an ammonium-derived metabolite, at least in plant species with lower nitrogen requirements, or in the form of amides, typical of legumes such as pea (Gu *et al.*, 2022).

The amount of ammonium-derived ^15^N translocated to the shoot was enhanced at a slightly acidic pH and was significantly higher in pea than in spinach at the highest nitrogen dose (10 mM; Figs 9, 10), which could be attributed to the higher number of AMT2-type proteins present in pea. Indeed, pea contains five AMT2-type proteins, four more than in spinach and Arabidopsis (Supplementary Table S2; Supplementary Fig. S3), whose functions are mainly ammonium transport from root to shoot under high ammonium nutrition (Giehl *et al.*, 2017), but also in the arbuscular mycorrhizal symbiosis in some species (Breuillin-Sessoms *et al.*, 2015). A similar trend was observed for translocation of ^15^N derived from nitrate (Fig. 9G–L), so we cannot exclude the possibility that a higher number of NPF-like proteins in pea (69 compared with 57 in spinach or 53 in Arabidopsis; Supplementary Table S2; Supplementary Fig. S1) confer to pea plants a greater capacity for long-distance transport of nitrate derivatives such as amides (Christensen and Jochimsen, 1983). Interestingly in spinach, the decrease in the ^15^N translocation derived from ammonium at pH 8 could be due to its lower repertoire of AMT2-like proteins, whose transport is electroneutral (i.e. uncoupling NH_3_ from H^+^), thus affecting the capacity of ammonium transport from root to shoot under more alkaline conditions (Neuhäuser *et al.*, 2009).

### Nitrate-linked hormone balance confirms pH and nitrogen dependence of ammonium sensitivity in spinach and pea

In assessing hormones as potential indicators of the nitrogen source’s impact on plant growth, the presence of nitrate notably enhances the accumulation of IAA in spinach roots (Fig. 11). This aligns with the documented local biosynthesis of auxins by nitrate (Jia *et al.*, 2021). The higher accumulation in roots at pH 6 compared with pH 8 could be explained by a higher apoplastic flux of protonated IAA, as indicated by the probable higher shoot acidification under sole ammonium treatment. On the other hand, the lower IAA content in spinach roots under sole ammonium treatment could be in line with Kudoyarova *et al*. (1997) who observed a lower IAA content in wheat roots supplied with ammonium compared with nitrate. These results suggest that lower content and distribution of IAA to the roots may somehow contribute to root inhibition by ammonium in nitrate-preferring species.

Interpreting individual changes in IAA and CK levels from plant extracts can be challenging, but examining their ratios provides a comprehensive perspective and indicates potential imbalances in IAA and CK biosynthesis for plant growth. The well-known homeostatic feedback loop governing the coordinated and fine-tuned biosynthesis of CK and IAA involves balanced levels of both hormones and their signaling actions (Jones *et al.*, 2010). Specifically, elevated IAA levels lead to the down-regulation of CK biosynthesis, and vice versa, with CK levels influencing IAA biosynthesis, particularly in young tissues (Jones *et al.*, 2010). This study highlights a notable increase in the IAA to CK ratio induced by nitrate for both species. Consistent with Jones *et al.* (2010), nitrate seems to play a crucial role in stimulating the ‘homeostasis feedback loop’, regulating the relative levels of IAA and CK in plant tissues, ultimately promoting plant growth and development.

Unexpectedly, JAs, recognized for their role in regulating the expression of numerous stress-responsive genes and promoting specific protective mechanisms (Li *et al.*, 2018), demonstrated a distinct reliance on accumulation in both spinach roots and shoots under exclusive nitrate nutrition, especially at pH 6. This observed pattern corresponds to the noted close association between JAs and nitrate transporters, potentially strengthened at lower pH when nitrate transport capacity is induced, consequently leading to increased JA accumulation. Indeed, some NPF proteins, such as NPF2.10/GTR1 expressed in *Xenopus* oocytes, show the ability to transport the bioactive form JA-Ile after its application to the growth medium (Saito *et al.*, 2015). However, this reasoning is not consistent with our observations, because pea plants, which have a higher number of NPF-type transporters, do not show higher levels of JA. Therefore, it remains to be evaluated how the distribution and repertoire of NPF for each species, with the wide range of substrates, influences the transport and signaling of JA/JA-Ile *in planta*.

On the contrary, treatments containing ammonium showed a distinct and pH-dependent response to systemic disturbances, inducing a coordinated accumulation of indicators such as ABA, SA, the glycolytic by-product MG, and the recruitment of *cis* over *trans* forms of CK, suggesting that they are reliable indicators of ammonium toxicity for spinach and pea plants.

Finally, the *c*ZR to *t*ZR ratio has been described as an early indicator of senescence processes (Silva-Navas *et al.*, 2019). The higher values of the *c*ZR to *t*ZR ratio shown under the stressful treatments, pH–nitrogen source combination for each species, support the proposal to use it as an indicator of senescence processes even before the observation of the phenotype exhibited.

### Concluding remarks

Collectively, the analyses of biomass, nutrient status, nitrogen uptake capacities, and hormone levels suggest that the evolutionary adaptation that plant species have undergone in the habitats in which they have survived has characterized them in their ability to acquire different sources of nitrogen by providing them with different repertoires of nitrate and ammonium transporters, as well as in their ability to grow in different pH ranges, among others. Therefore, plant species with higher tolerance to ammonium are associated with fewer *NRT2*-type genes, more *AMT2*-type genes, and are linked to acidic soils, lower nitrogen demand, and lower spread capacity. Thus, the varying sensitivity to ammonium primarily stems from the distinct ‘need’ for nitrate. Finally, this study leads to the following conclusions, answering the three main questions proposed herein to achieve the main objective of ‘Identifying the determining factors that explain the nitrogen uptake capacities and the sensitivity to the main nitrogen forms’.

(i) The most influential ecophysiological factors are soil pH and nitrate requirement, which are closely related to the number of genes encoding NRT2-type transporters. Acidic soil conditions limit nitrate availability, causing species adapted to such soils to have a weaker preference for nitrate and a less diverse repertoire of high-affinity transporters, making them less susceptible to nitrate deficiency or ammonium abundance. In addition, more ammonium-tolerant species tend to have an increased number of genes for AMT2-type transporters.
(ii) Ammonium uptake capacity differs between ammonium-tolerant and ammonium-sensitive species. In the categorically divergent examples, on one hand, spinach is critically dependent on nitrate not only to partially mitigate ammonium toxicity by inhibiting ammonium uptake but also to enhance its growth, especially at alkaline pH. On the other hand, pea shows greater resilience to different ammonium concentrations, benefiting from a slightly acidic pH that could improve phosphorus acquisition and the translocation of nitrogen derivatives through extended homologs of AMT2 and NPF transporters.
(iii) Different ammonium sensitivities can be predicted and detected by hormonal and/or metabolic indicators. Significant increases in the *c*ZR to *t*ZR ratio and MG content in both shoot and root, ABA and SA especially in the shoot, and decreases in IAA and IAA to CKs ratio in roots can be detected before or simultaneously with toxicity symptoms aggravated by pH.

## Supplementary data

The following supplementary data are available at *JXB* online.

Table S1. References to the available studies published up to March 2022.

Table S2. Mean values of some of the ecological characteristics of the meta-analyzed plant species and their number of nitrogen transporter genes.

Fig. S1. Growth of pea and spinach under strict ammonium nutrition at pH 6 and 8.

Fig. S2. Ammonium and nitrate contents in plant tissues of spinach and pea plants grown with different ammonium–nitrate proportions at pH 6 and 8.

Fig. S3. Content of soluble cations in plant tissues of spinach and pea plants grown with different ammonium–nitrate proportions at pH 6 and 8.

Fig. S4. Content of soluble anions in plant tissues of spinach and pea plants grown with different ammonium–nitrate proportions at pH 6 and 8.

Fig. S5. Comparison of ^15^N uptake between spinach and pea for each pH.

Fig. S6. Root to shoot translocation of ^15^N between spinach and pea for each pH.

Fig. S7. Phylogenetic tree of eight subfamilies of NRT1/PTR FAMILY (NPF) proteins from *Arabidopsis thaliana* L., *Spinacia oleracea* L., *Pisum sativum* L., and *Oryza sativa* L.

Fig. S8. Phylogenetic tree of NRT2- and NRT3-type high-affinity transporters for nitrate from *Arabidopsis thaliana* L., *Spinacia oleracea* L., *Pisum sativum* L., and *Oryza sativa* L.

Fig. S9. Phylogenetic tree of AMT1- and AMT2-type high-affinity transport proteins for ammonium from *Arabidopsis thaliana* L., *Spinacia oleracea* L., *Pisum sativum* L., and *Oryza sativa* L.

Dataset S1. Raw bootstrap data.

erae106_suppl_Supplementary_Dataset_S1

erae106_suppl_Supplementary_Tables_S1-S2

erae106_suppl_Supplementary_Figures_S1-S9

## Data Availability

All data necessary to evaluate the conclusions of this paper are included in the article and/or supplementary data, and are available in the public repository Dryad: https://doi.org/10.5061/dryad.gmsbcc2w6 (Rivero-Marcos *et al*., 2024). Answers to additional questions related to this study may be requested from the authors.
