# Supplementary material for: Plant ammonium sensitivity is associated with external pH adaptation, repertoire of nitrogen transporters, and nitrogen requirement
Source: J Exp Bot. 2024 Mar 11;75(11):3557–78. doi: 10.1093/jxb/erae106 (PMC11358259; doi:10.1093/jxb/erae106)
Supplement: erae106_suppl_Supplementary_Figures_S1-S9 [file erae106_suppl_supplementary_figures_s1-s9.pdf]

Table S1 and S2 (Excel format)

Table S1. References to the available studies published up to March 2022, from which the information of whole plant, root, and shoot dry biomass of different species fed with sole NH_4^+ *vs* NO_3^- was obtained for the calculation of LnBR.

Table S2. Mean values of some of the ecological characteristics of the meta-analyzed plant species and their number of nitrogen transporter genes. *N* is described as soil nitrophility/fertility and *R* as soil pH according to the 'Ellenberg' index, and *spread* as the number of countries in which the species is considered invasive according to the 'GBIF' database. Published reference is cited where the number of *NRT*s or *AMT*s were described for the indicated plant species. | Highlighted in yellow are the Ellenberg's indices described according to the Italian flora (Guarino et al., 2012), and in blue according to British flora (Hill et al., 1999). In green extrapolated according to GBIF and PFAF databases. Superscript 'T' refers to the number of nitrogen transport genes in orthology with Arabidopsis according to the 'TAIR' database; 'S' according to 'Spinach Base Genome'; and 'P' according to 'Pisum sativum Cameor' database.

Fig. S1

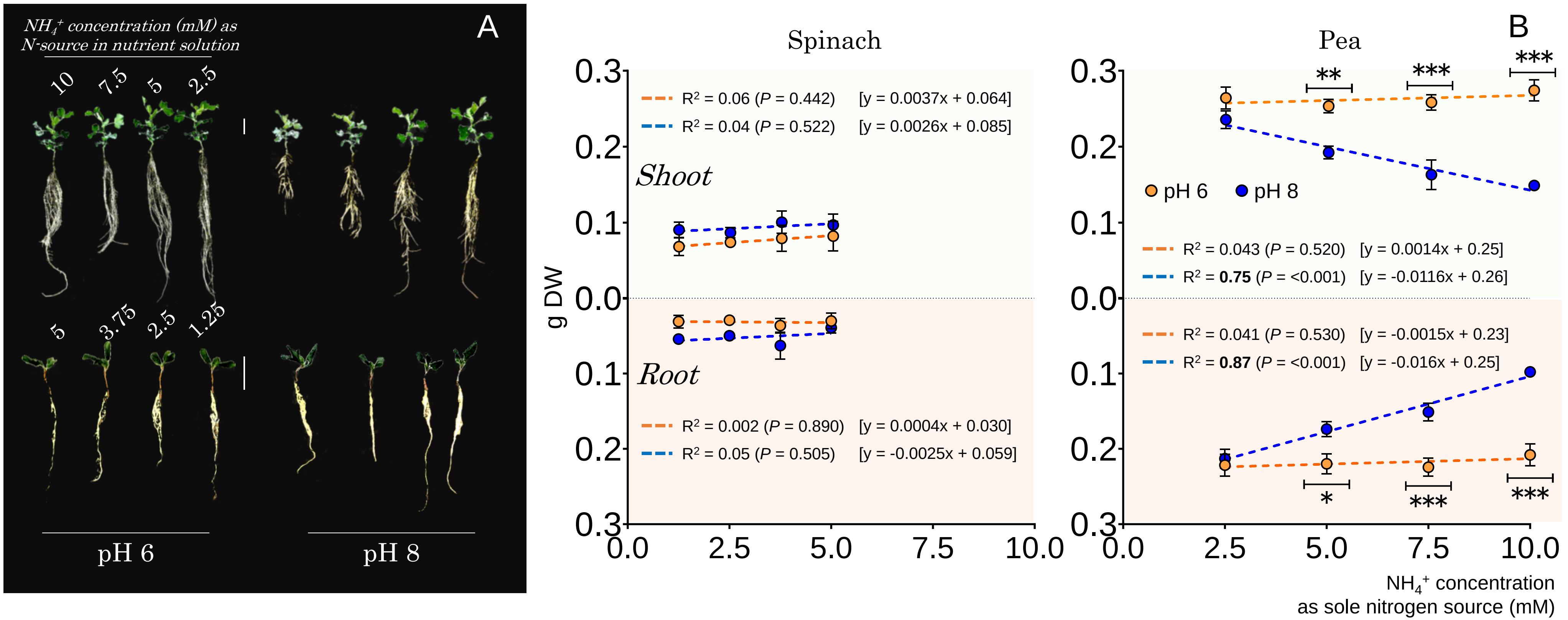

Fig. S1. Growth negatively affected by strict ammonium nutrition in spinach (pH independent) and aggravated in pea at pH 6. **A.** Phenotype of hydroponically-grown spinach and pea plants. White vertical lines indicate the scale (5 cm). **B.** Shoot and root biomass between pH 6 and 8. The dashed lines indicate the trend in the decrease in biomass as the concentration of ammonium increases in the nutrient solution, quantified by R^2, whose statistical significance is marked in bold ($P \leq 0.05$). Asterisks represent the differences in each pH depending on the treatment (t Student test). * = 0.05 > P > 0.01, ** = 0.01 > P > 0.001; *** = < 0.001. DW refers to dry weight. | Spinach and pea plants were grown for three and two weeks respectively with a continuous supply of ammonium $(NH_4)_2SO_4$ as the only source of nitrogen at the indicated concentrations to test the degree of ammonium sensitivity and select the maximum dose for a comparison of toxicity scenarios.

Fig. S2

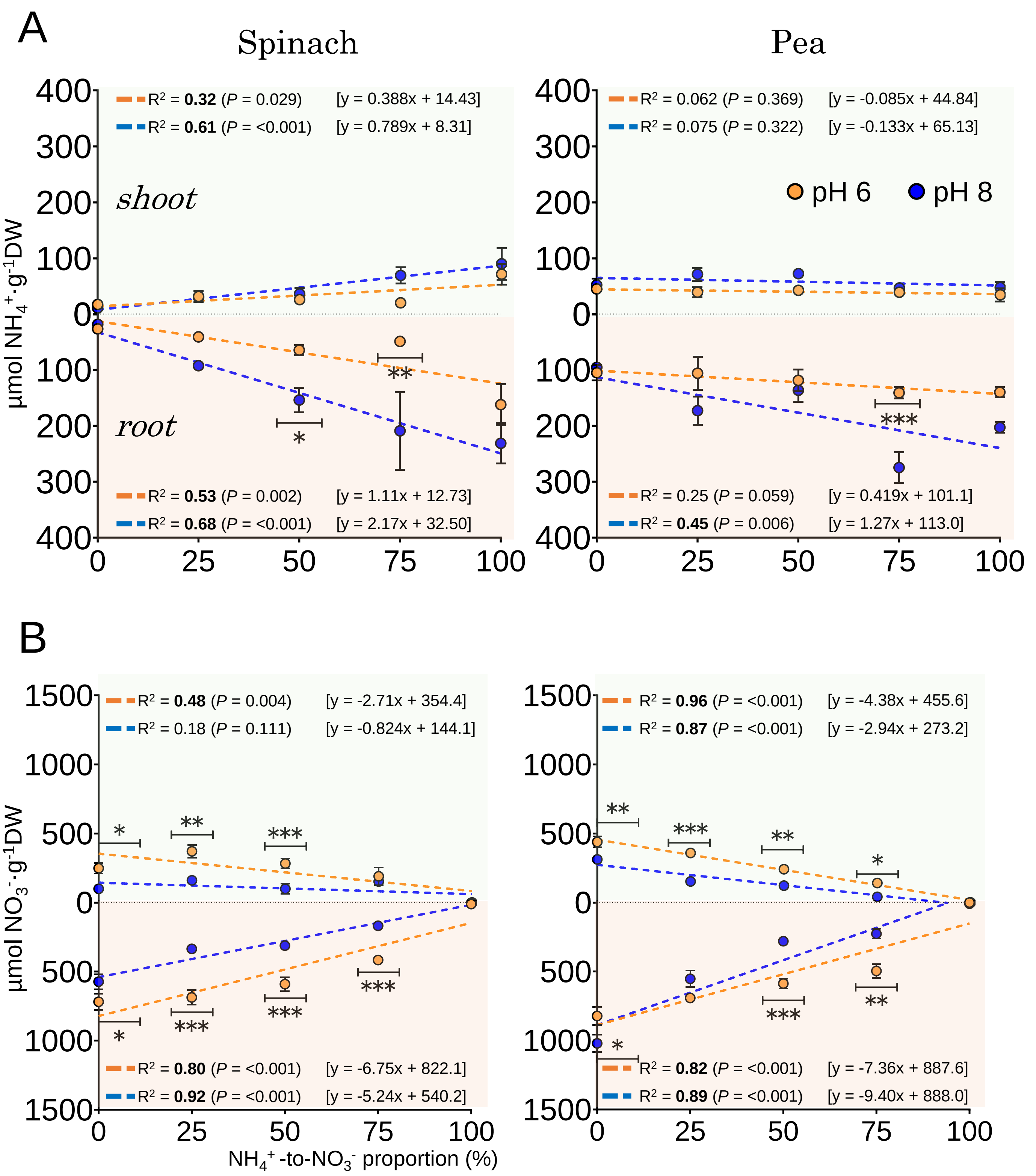

Fig. S2. Ammonium (A) and nitrate (B) content (µmol g^{-1} DW) in shoot and root of spinach and pea plants treated with NH_4^+-to-NO_3^- proportion from 0% (strict nitrate) to 100% (strict ammonium) at pH 6 and 8. Data represent means ± SE (n = 5). The dashed lines indicate the trend in the contents as the concentration of ammonium increases in the nutrient solution, quantified by R^2, whose statistical significance is marked in bold (*P* ≤ 0.05). Asterisks represent the differences in each pH depending on the treatment (t Student test). * = 0.05 > *P* > 0.01, ** = 0.01 > *P* > 0.001; *** = *P* < 0.001. DW refers to dry weight.

Fig. S3

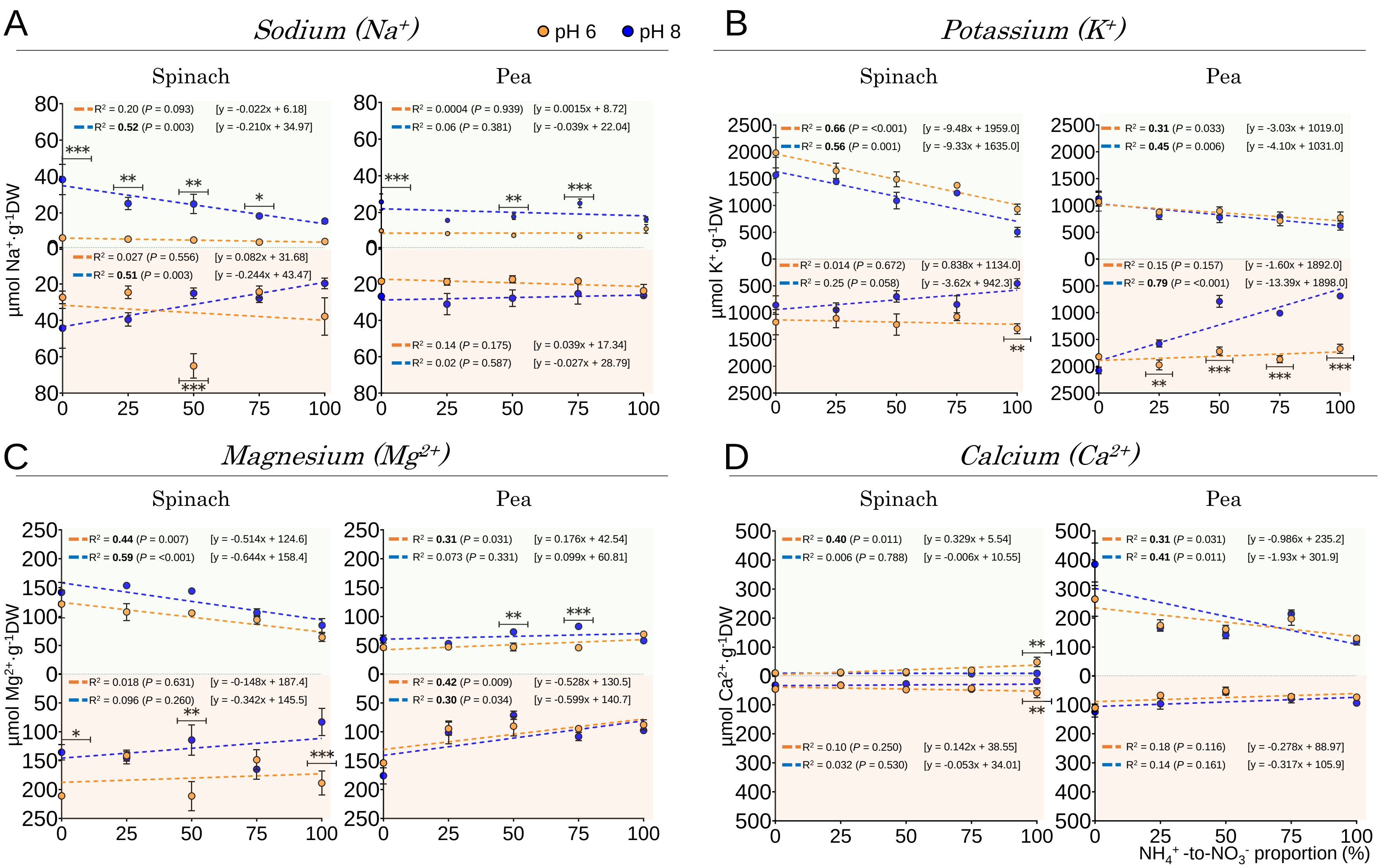

Fig. S3. Content of main soluble cations in shoot and root of spinach and pea plants treated with different NH_4^+-to-NO_3^- proportions at pH 6 and 8. Data represent means ± SE (n=3). The dashed lines indicate the trend in the cation content as the concentration of ammonium increases in the nutrient solutions, quantified by R^2, whose statistical significance is marked in bold ($P \leq 0.05$). Asterisks represent the differences in each pH depending on the treatment (t Student test). * = 0.05 > P > 0.01, ** = 0.01 > P > 0.001; *** = < 0.001. DW refers to dry weight and NH_4^+-to-NO_3^- proportion range from 0% (strict NO_3^-) to 100% (strict NH_4^+). Total nitrogen was 5 mM for spinach and 10 mM for pea.

Fig. S4

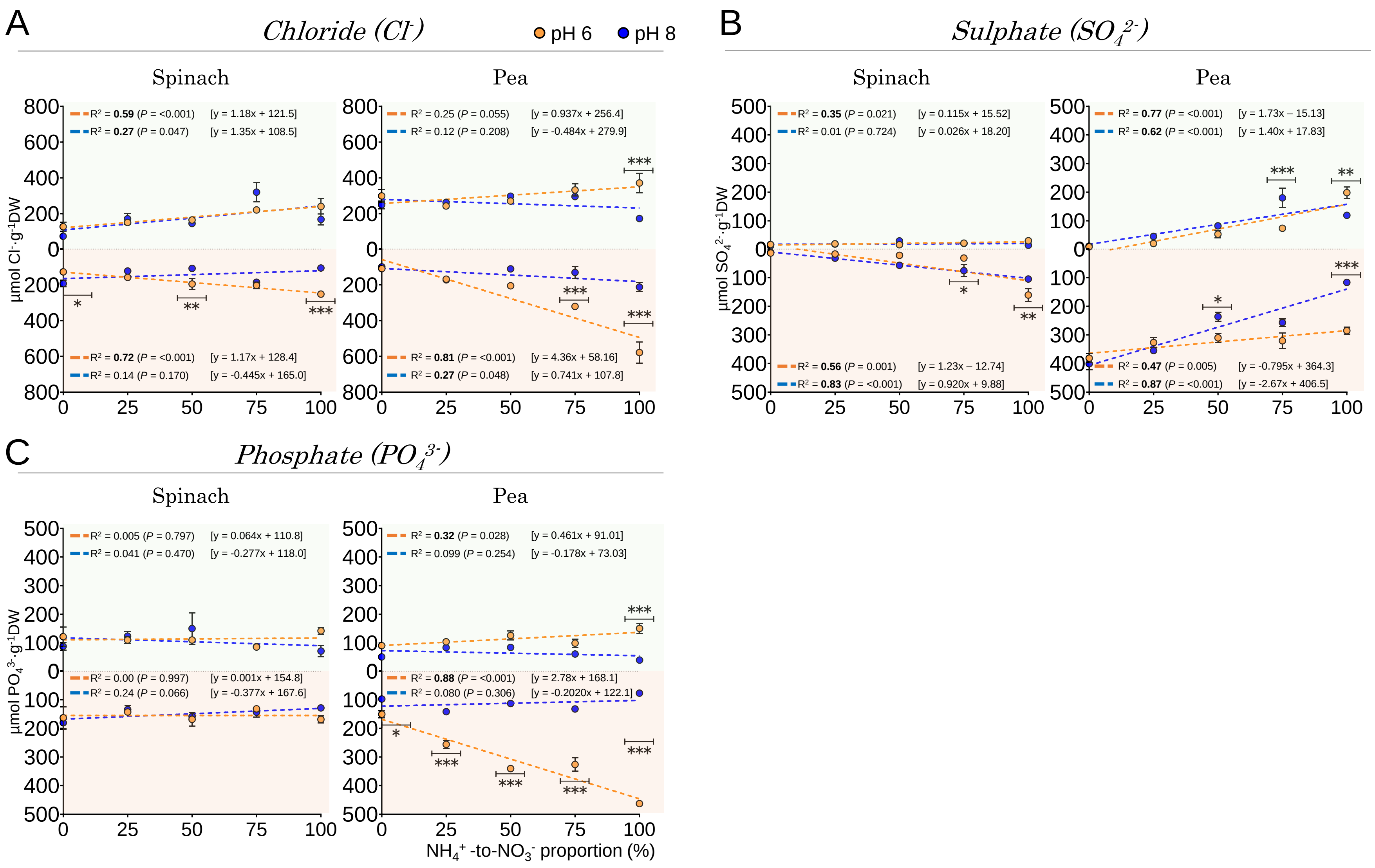

Fig. S4 Content of main soluble anions in shoot and root of spinach and pea plants treated with different NH_4^+-to-NO_3^- proportions at pH 6 and 8. Data represent means ± SE (n = 3). The dashed lines indicate the trend in the cation content as the concentration of ammonium increases in the nutrient solutions, quantified by R^2, whose statistical significance is marked in bold ($P \leq 0.05$). Asterisks represent the differences in each pH depending on the treatment (t Student test). * = 0.05 > P > 0.01, ** = 0.01 > P > 0.001; *** = < 0.001. DW refers to dry weight and NH_4^+-to-NO_3^- proportion range from 0% (strict NO_3^-) to 100% (strict NH_4^+). Total nitrogen was 5 mM for spinach and 10 mM for pea.

Fig. S5

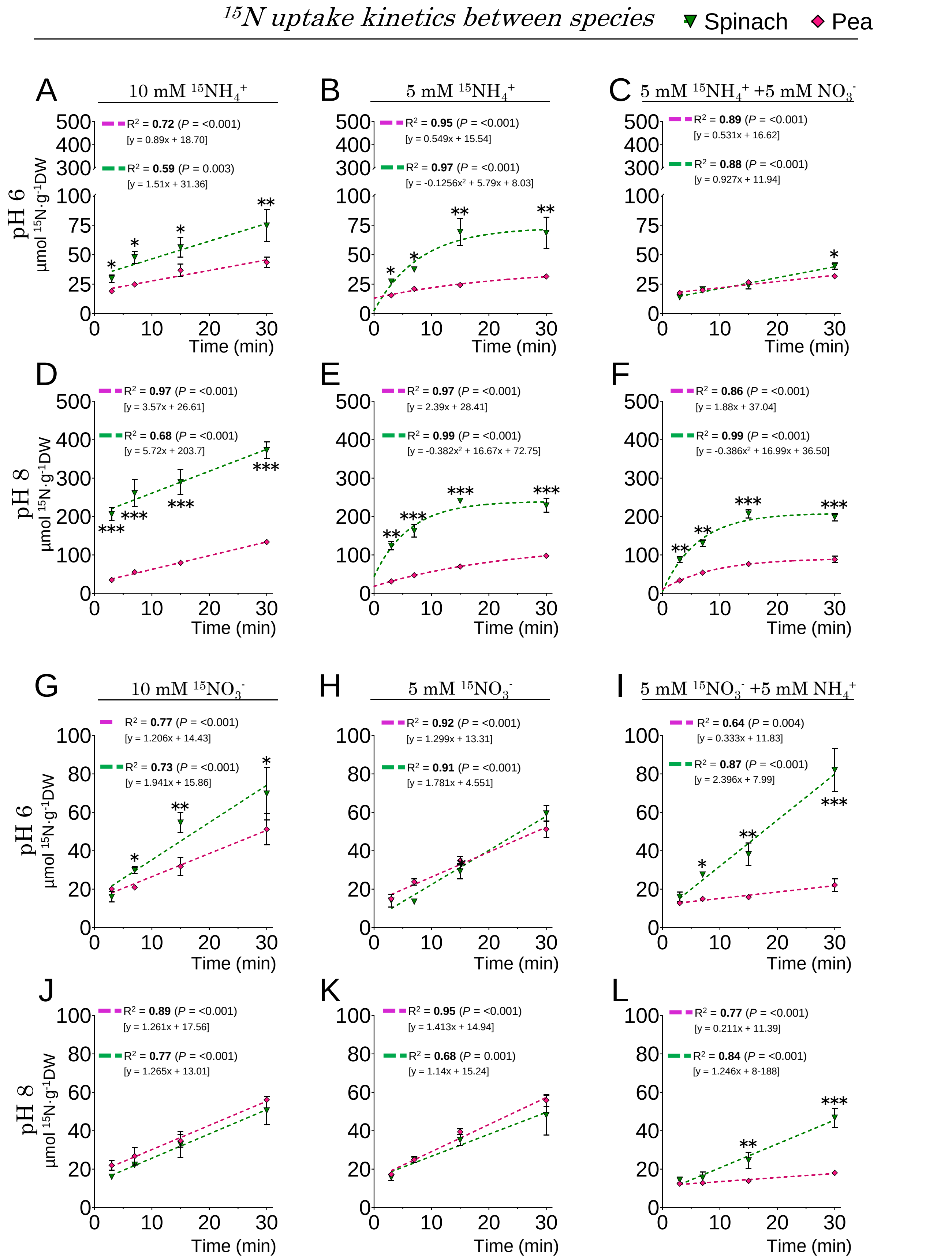

Fig. S5. Comparison of ^{15}N uptake between spinach and pea for each pH. A to F, influx of ^{15}N-labelled NH_4^+ (**A to F**) or ^{15}N-labelled NO_3^- (**G to L**) into roots over a 30 min incubation at 10- and 5-mM concentrations at pH 6 and 8. The data represents the means ± SE (n = 3-5). The dashed lines indicate the trend in the ^{15}N content throughout the incubation time, quantified by R^2, whose statistical significance is marked in bold ($P \leq 0.05$). Asterisks represent the differences between species for each time point (t Student test). * = 0.05 > *P* > 0.01, ** = 0.01 > *P* > 0.001; *** = < 0.001. DW refers to dry weight.

Fig. S6

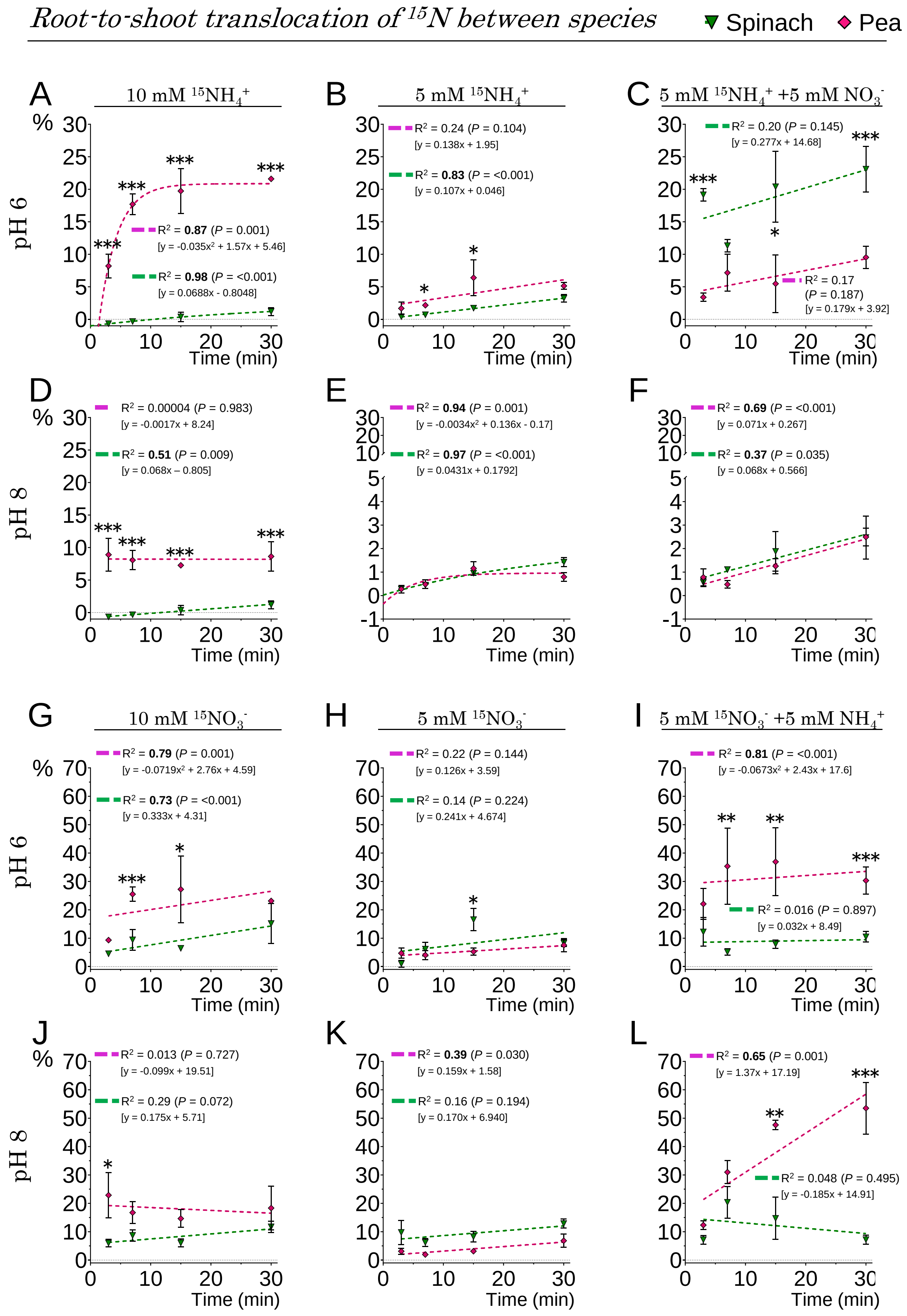

Fig. S6. Root-to-shoot translocation of ^{15}N between spinach and pea for each pH. A to F, percentage of ^{15}N-labelled NH_4^+ (**A to F**) or ^{15}N-labelled NO_3^- (**G to L**) translocated in shoot over a 30 min incubation at 10- and 5-mM concentrations at pH 6 and 8. The data represents the means ± SE (n = 3-5). The dashed lines indicate the trend in in the ^{15}N content in shoot with respect to the total plant throughout the incubation time, quantified by R^2, whose statistical significance is marked in bold ($P \leq 0.05$). Asterisks represent the differences between species for each time point (t Student test). * = 0.05 > P > 0.01, ** = 0.01 > P > 0.001; *** = < 0.001. DW refers to dry weight.

Fig. S7

Phylogenetic tree of NRT1/ PTR FAMILY (NPF) proteins

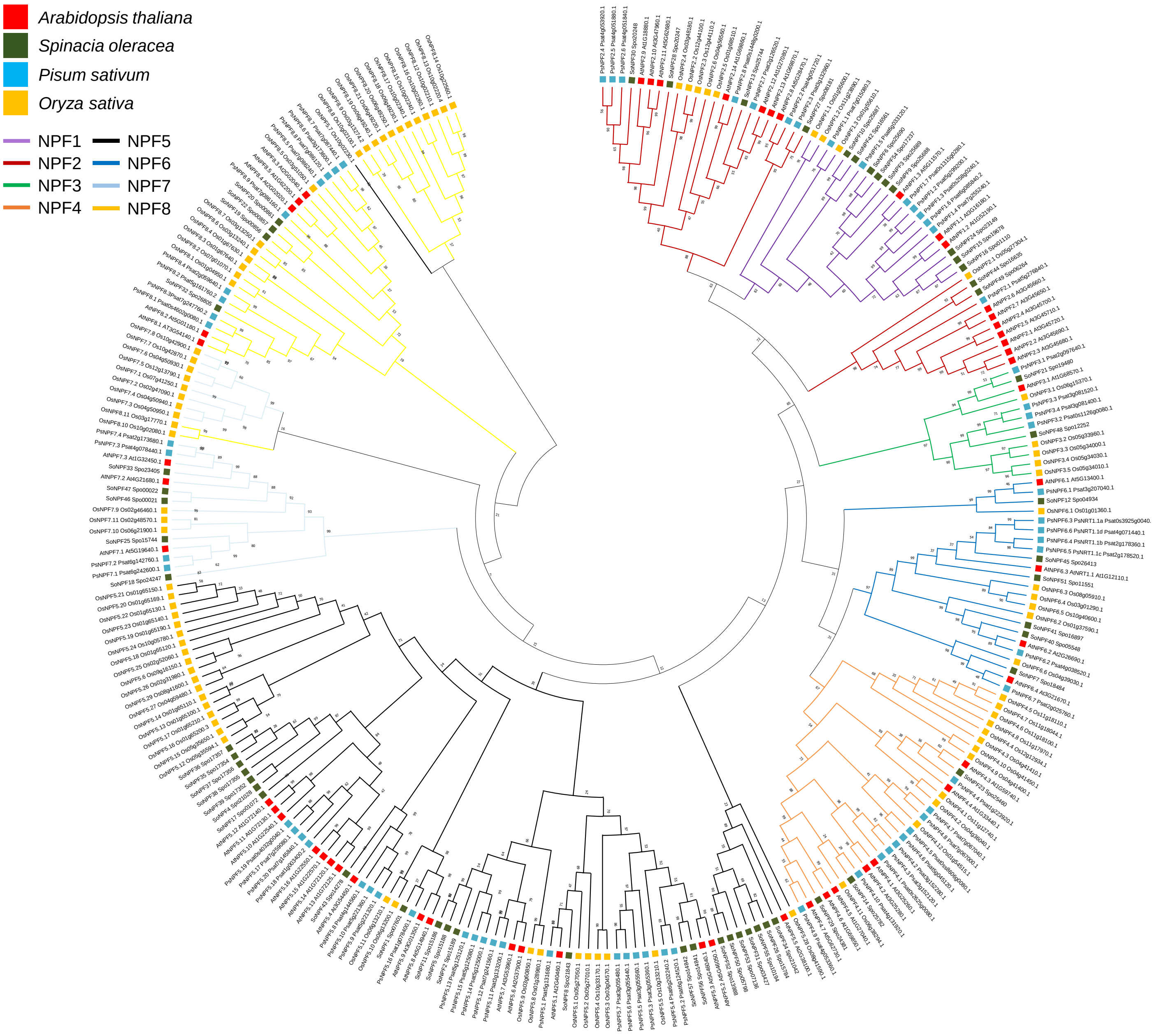

Fig. S7. Phylogenetic tree of NRT1/ PTR FAMILY (NPF) proteins from *Arabidopsis thaliana*, *Spinacia oleracea*, *Pisum sativum* and *Oryza sativa*. Eight NPF families are indicated. The evolutionary history was inferred by using the Maximum Likelihood method and JTT matrix-based model. The tree with the highest log likelihood (-194116.77) is shown. The percentage of trees in which the associated taxa clustered together is shown below the branches. Initial tree(s) for the heuristic search were obtained automatically by applying Neighbor-Join and BioNJ algorithms to a matrix of pairwise distances estimated using the JTT model, and then selecting the topology with superior log likelihood value. The tree is drawn to scale, with branch lengths measured in the number of substitutions per site. This analysis involved 272 amino acid sequences and there was a total of 1417 positions in the final dataset. Evolutionary analyses were conducted in MEGA11.

Fig. S8

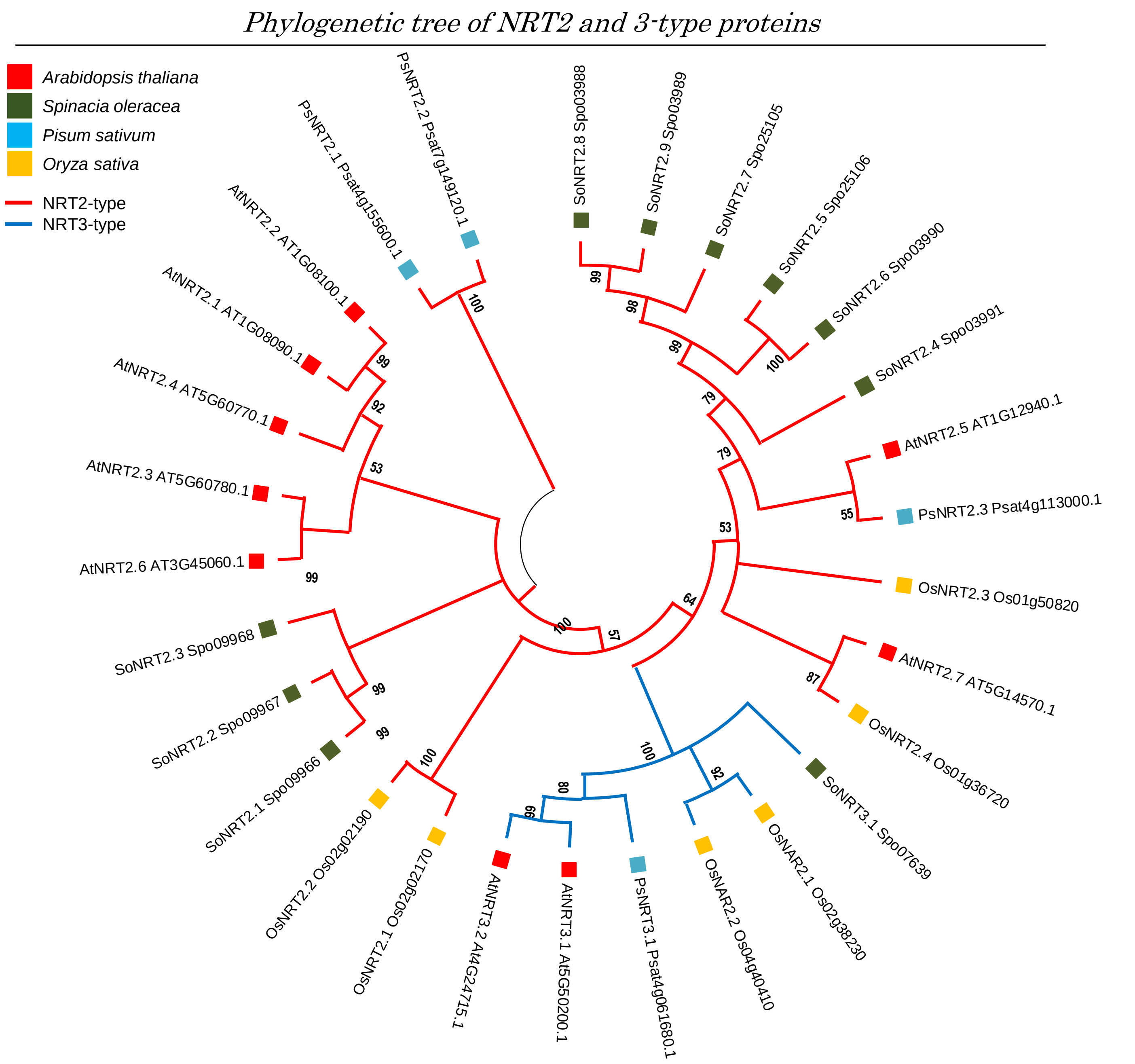

Fig. S8. Phylogenetic tree of NRT2- and NRT3-type high-affinity transport proteins for nitrate from *Arabidopsis thaliana*, *Spinacia oleracea*, *Pisum sativum* and *Oryza sativa*. The evolutionary history was inferred by using the Maximum Likelihood method and JTT matrix-based model. The tree with the highest log likelihood (-14524.83) is shown. The percentage of trees in which the associated taxa clustered together is shown below the branches. Initial tree(s) for the heuristic search were obtained automatically by applying Neighbor-Join and BioNJ algorithms to a matrix of pairwise distances estimated using the JTT model, and then selecting the topology with superior log likelihood value. The tree is drawn to scale, with branch lengths measured in the number of substitutions per site. This analysis involved 29 amino acid sequences and there was a total of 573 positions in the final dataset. Evolutionary analyses were conducted in MEGA11.

Fig. S9

Phylogenetic tree of AMT proteins

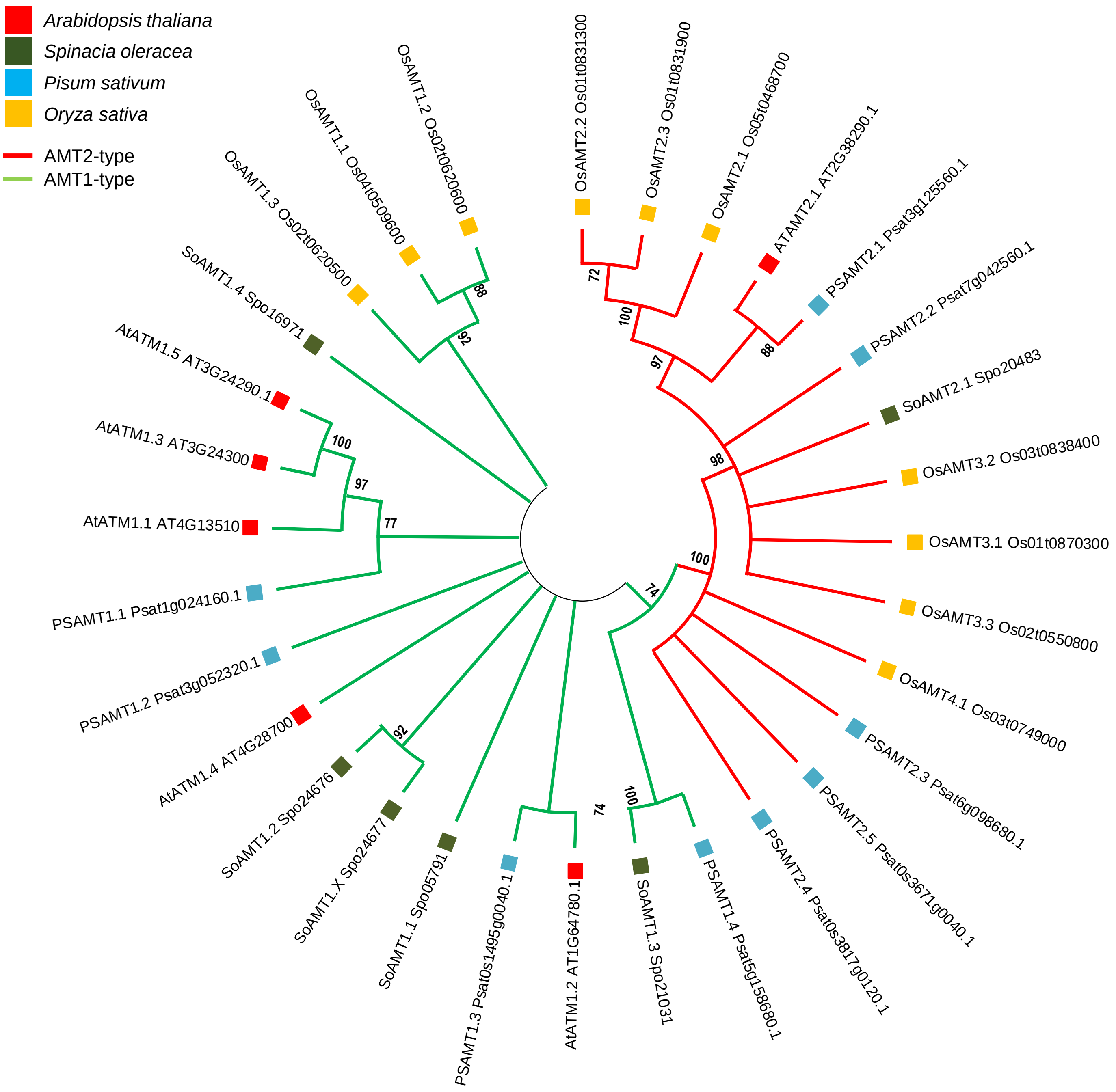

Fig. S9. Phylogenetic tree of AMT1- and AMT2-type high-affinity transport proteins for ammonium from *Arabidopsis thaliana*, *Spinacia oleracea*, *Pisum sativum* and *Oryza sativa*. An unrooted neighbour-joining tree and bootstrap analysis were performed with the MEGAX program. The evolutionary history was inferred by using the Maximum Likelihood method and JTT matrix-based model. The tree with the highest log likelihood (-17830.22) is shown. The percentage of trees in which the associated taxa clustered together is shown below the branches. Initial tree(s) for the heuristic search were obtained automatically by applying Neighbor-Join and BioNJ algorithms to a matrix of pairwise distances estimated using the JTT model, and then selecting the topology with superior log likelihood value. The tree is drawn to scale, with branch lengths measured in the number of substitutions per site. This analysis involved 31 amino acid sequences and there was a total of 582 positions in the final dataset. Evolutionary analyses were conducted in MEGA11.
